# Metagenomic characterization of gut microbiota in rheumatoid arthritis-associated interstitial lung disease: taxonomic shifts and clinical correlations

**DOI:** 10.3389/fimmu.2026.1868704

**Published:** 2026-06-12

**Authors:** Ran Fan, Qifeng Zang, Yan Xu, Ling Gao, Jun Zhou, Yinshan Zang

**Affiliations:** 1 Department of Rheumatology and Immunology, Jiangsu Province (Suqian) Hospital, Suqian, Jiangsu, China; 2 School of Life Sciences, Bengbu Medical University, Bengbu, Anhui, China; 3 Department of Rheumatology and Immunology, Suqian First Hospital, Suqian, Jiangsu, China

**Keywords:** Escherichia/Shigella, faecalibacterium, gut microbiome, gut-lung axis, interstitial lung disease, metagenomics, rheumatoid arthritis

## Abstract

**Background:**

Rheumatoid arthritis-associated interstitial lung disease (RA-ILD) is a severe extra-articular manifestation with limited diagnostic biomarkers. While gut microbiota dysbiosis contributes to rheumatoid arthritis (RA) pathogenesis, its specific role in RA-ILD remains poorly characterized.

**Methods:**

We performed shotgun metagenomic sequencing on fecal samples from 41 participants: 10 RA-ILD patients, 20 RA patients without ILD (RA-non-ILD), and 11 healthy controls (HCs). We assessed alpha and beta diversity, differential abundance (Wilcoxon rank-sum tests with FDR correction), Spearman correlations with clinical parameters, microbial co-occurrence networks, and random forest classification.

**Results:**

Alpha and beta diversity did not differ significantly among groups. After FDR correction, no genus differed significantly between RA-ILD and RA-non-ILD. Exploratory analysis (uncorrected *P* < 0.05) revealed enrichment of *Escherichia/Shigella* in RA-ILD (11.72% vs. 2.66%, *P* = 0.003) and depletion of *Roseburia* (1.05% vs. 3.77%, *P* = 0.005) and *Ruminococcus* (5.98% vs. 7.85%, *P* = 0.032), while *Faecalibacterium* showed a trend toward depletion without reaching nominal significance (4.45% vs. 4.66%, *P* = 0.409). Correlation analysis revealed a dichotomous pattern: pro-inflammatory genera correlated positively with disease activity, while butyrate-producing genera correlated negatively. Co-occurrence network analysis showed RA patients had a more complex network than HC and RA-ILD. Random forest classification identified *Bifidobacterium*, unclassified_ Oscillospiraceae, and unclassified_Lachnospiraceae as top discriminators between HC and RA, and unclassified_ Bacteroidaceae, *Parabacteroides*, and *Blautia* for RA-ILD vs RA.

**Conclusions:**

RA-ILD is associated with specific gut microbial alterations—notably *Escherichia/Shigella* enrichment and depletion of *Roseburia* and *Ruminococcus*—despite preserved overall diversity. These changes correlate with systemic inflammation and suggest a role for the gut microbiota in RA-ILD pathogenesis via the gut-lung axis. The identified taxa warrant validation as candidate biomarkers in larger cohorts.

## 1 Introduction

Rheumatoid arthritis (RA) is a chronic systemic autoimmune disorder affecting approximately 0.5% to 1% of the global population. The disease is characterized by progressive synovitis that leads to joint destruction and disability (1). Beyond articular involvement, RA manifests with diverse extra-articular complications, among which interstitial lung disease (ILD) represents one of the most frequent and clinically consequential (2). Epidemiological studies indicate that 10% to 20% of RA patients develop clinically significant ILD during their disease course. Subclinical pulmonary involvement detected by high-resolution computed tomography (HRCT) may occur in up to 50% to 60% of patients (3, 4). RA-ILD substantially worsens prognosis and contributes to increased morbidity and mortality. Median survival following diagnosis ranges from 3 to 8 years, a prognosis worse than that of idiopathic pulmonary fibrosis (5, 6).

The pathogenesis of RA-ILD remains incompletely understood. Genetic susceptibility factors, including the MUC5B promoter polymorphism, have been implicated (7). Environmental triggers, particularly smoking, are well-established risk factors (8). However, the mechanisms linking systemic autoimmunity to pulmonary fibrosis remain elusive, and clinically useful biomarkers for early RA-ILD detection are lacking. Current diagnostic approaches rely heavily on HRCT and pulmonary function testing, which may detect disease only after substantial parenchymal damage has occurred (9). Serum biomarkers routinely used in RA management, including rheumatoid factor (RF) and anti-citrullinated protein antibodies (ACPAs), correlate poorly with ILD presence or progression (10). This diagnostic gap underscores the urgent need for novel, non-invasive biomarkers to enable earlier intervention and improved outcomes.

The gut microbiome has emerged as a critical modulator of systemic immunity, with its composition and function increasingly recognized as influential factors in autoimmune disease pathogenesis (11, 12). The “gut-joint axis” concept, supported by extensive preclinical and clinical evidence, posits that intestinal dysbiosis contributes to RA development. Proposed mechanisms include intestinal barrier disruption, translocation of microbial products, and dysregulation of T-cell subsets particularly the balance between pro-inflammatory Th17 cells and anti-inflammatory regulatory T cells (Tregs) (13, 14). Metagenomic studies have consistently demonstrated that RA patients exhibit reduced gut microbial diversity. They show depletion of short-chain fatty acid (SCFA)-producing commensals such as *Faecalibacterium prausnitzii* and enrichment of potentially pro-inflammatory taxa including *Prevotella copri* (15, 16). Furthermore, fecal microbiota transplantation from RA patients into germ-free mice can recapitulate arthritis susceptibility, providing causal evidence for microbiome involvement (17).

Beyond articular disease, accumulating evidence supports the existence of a “gut-lung axis” bidirectional communication between intestinal and pulmonary mucosal immune systems (18, 19). Gut-derived microbial metabolites, particularly SCFAs, enter the circulation and modulate pulmonary immune responses, influencing susceptibility to allergic airway inflammation and infection (20). Conversely, respiratory infections can alter gut microbiota composition, highlighting the interconnectedness of these mucosal sites (21). In the context of fibrotic lung disease, animal studies demonstrate that antibiotic-induced gut dysbiosis exacerbates bleomycin-induced pulmonary fibrosis, while probiotic administration exerts protective effects (22, 23). Patients with systemic sclerosis, another autoimmune condition frequently complicated by ILD, exhibit distinct gut microbial signatures associated with the severity of pulmonary involvement (24).

Despite this converging evidence implicating the gut-lung axis in pulmonary fibrosis and the established role of gut microbiota in RA pathogenesis, the gut microbiome of RA-ILD patients has received limited investigation. A recent multi-kingdom metagenomic study by Xing and colleagues compared RA-ILD patients (n=30) with RA-non-ILD patients (n=30) and healthy controls (HCs). Their work revealed alterations across bacterial, fungal, and viral components (25). They identified depletion of butyrate-producing bacteria including *Faecalibacterium prausnitzii* and enrichment of certain *Ruminococcus* species in RA-ILD, alongside shifts in fungal and viral communities. However, replication of these findings in independent cohorts and exploration of associations with detailed clinical parameters remain important next steps.

We therefore designed the present study to characterize the gut microbiome in RA-ILD patients using shotgun metagenomic sequencing, with three specific objectives. First, to compare microbial diversity and community structure among RA-ILD patients, RA-non-ILD patients, and healthy controls. Second, to identify specific taxonomic features distinguishing RA-ILD from RA-non-ILD. Third, to examine associations between microbial features and clinically relevant parameters including inflammatory markers (ESR, CRP) and autoantibody status (RF, anti-CCP). We hypothesized that RA-ILD would be associated with distinct gut microbial alterations, particularly involving depletion of SCFA-producing commensals and enrichment of pro-inflammatory taxa, which would correlate with systemic inflammation.

## 2 Materials and methods

### 2.1 Study design and participants

This cross-sectional observational study was conducted at the Department of Rheumatology and Immunology, Suqian First Hospital, between July 2023 and December 2024. The study protocol was approved by the Ethics Committee of Suqian First Hospital (approval number: 2022071). Written informed consent was obtained from all participants prior to enrollment. All procedures adhered to the principles of the Declaration of Helsinki.

We enrolled 41 participants comprising three groups. The RA-ILD group included 10 patients fulfilling the 2010 American College of Rheumatology/European League Against Rheumatism (ACR/EULAR) classification criteria for RA (26) with ILD confirmed by HRCT. ILD diagnosis required the presence of typical findings including reticular opacities, traction bronchiectasis, honeycombing, or ground-glass opacities with a usual interstitial pneumonia (UIP) or non-specific interstitial pneumonia (NSIP) pattern. A radiologist and rheumatologist independently interpreted all HRCT images. The RA-non-ILD group included 20 patients meeting RA classification criteria with no clinical evidence of ILD and no abnormalities suggestive of ILD on chest HRCT performed within 6 months prior to enrollment. The healthy control (HC) group included 11 age- and sex-matched individuals without any history of autoimmune, inflammatory, or chronic pulmonary diseases. These individuals were recruited from the hospital’s medical examination center.

Inclusion criteria for RA patients were age ≥ 18 years, definite diagnosis of RA according to 2010 ACR/EULAR criteria, and stable disease-modifying antirheumatic drug (DMARD) regimen for at least 3 months prior to enrollment. Dose adjustments less than 50% of stable dose were permitted.

Exclusion criteria for all participants included antibiotic, probiotic, or prebiotic use within 3 months prior to fecal sampling. We also excluded individuals with systemic glucocorticoid use exceeding prednisone 10 mg/day or equivalent within 1 month. Other exclusion criteria were history of gastrointestinal surgery (except appendectomy or cholecystectomy), chronic gastrointestinal diseases (inflammatory bowel disease, celiac disease, chronic diarrhea), active infection at time of sampling, malignancy within the past 5 years, current smoking, alcohol abuse, and inability to provide informed consent.

This is an exploratory pilot study with a sample size determined by feasibility rather than formal power calculation. The modest sample size (especially n=10 for RA-ILD) limits statistical power for subgroup analyses and detection of small effect sizes; therefore, all findings should be interpreted as hypothesis-generating.

### 2.2 Clinical data collection

Demographic and clinical data were collected from all participants at enrollment. Laboratory parameters included erythrocyte sedimentation rate (ESR, mm/h), C-reactive protein (CRP, mg/L), rheumatoid factor (RF, IU/mL), and anti-cyclic citrullinated peptide (anti-CCP) antibodies (RU/mL). RF and anti-CCP were categorized as positive or negative according to manufacturer-recommended thresholds (RF ≥ 20 IU/mL; anti-CCP ≥ 25 RU/mL). For RA patients, additional disease-specific parameters were recorded. These included disease duration (months since diagnosis) and disease activity scores: Disease Activity Score in 28 joints (DAS28)-ESR, DAS28-CRP, Simplified Disease Activity Index (SDAI), and Clinical Disease Activity Index (CDAI). Current and prior disease-modifying anti-rheumatic drugs (DMARDs) use (methotrexate, hydroxychloroquine, leflunomide, sulfasalazine, biologics, targeted synthetic DMARDs) was documented.

### 2.3 Fecal sample collection and processing

Participants provided fresh morning stool samples using sterile collection containers. Samples were collected within 24 hours of admission and processed within 2 hours of passage. Using sterile spatulas, approximately 1 g of feces was aliquoted into 2 mL cryovials, snap-frozen in liquid nitrogen for 15 minutes, and subsequently stored at −80 °C until DNA extraction. To minimize freeze-thaw cycles, samples were aliquoted in duplicate.

### 2.4 DNA extraction

Total genomic DNA was extracted from approximately 200 mg of each fecal sample using the TIANamp Magnetic DNA Kit (Tiangen Biotech, Beijing, China) according to the manufacturer’s protocol. The extraction procedure included mechanical lysis by bead-beating, proteinase K digestion, and magnetic bead-based purification. DNA quality was assessed by 1% agarose gel electrophoresis and spectrophotometry (NanoDrop 2000, Thermo Fisher Scientific). DNA concentration was quantified using the Qubit dsDNA HS Assay Kit with a Qubit 4.0 Fluorometer (Thermo Fisher Scientific). Samples with OD260/280 ratios between 1.8 and 2.0 and DNA yield ≥ 1 μg were used for library preparation.

### 2.5 Library construction and metagenomic sequencing

Sequencing libraries were prepared using the NEBNext Ultra DNA Library Prep Kit for Illumina (New England Biolabs, Ipswich, MA, USA) following manufacturer instructions. Briefly, 1 μg of DNA per sample was sheared to approximately 350 bp fragments using ultrasonication (Covaris, Woburn, MA, USA). Fragmented DNA underwent end-repair, A-tailing, and ligation with Illumina-compatible adapters containing sample-specific barcodes. Adapter-ligated fragments were purified using AMPure XP beads (Beckman Coulter, Brea, CA, USA) and amplified by PCR. Library quality was assessed using the Agilent 2100 Bioanalyzer (Agilent Technologies, Santa Clara, CA, USA), and library concentration was quantified by quantitative PCR.

Pooled libraries were sequenced on the Illumina NovaSeq 6000 platform (Illumina, San Diego, CA, USA) using 2 × 150 bp paired-end chemistry. Sequencing was performed with a target of 10 Gb of clean data per sample.

### 2.6 Bioinformatic analysis

#### 2.6.1 Preprocessing and quality control

Raw sequencing reads in FASTQ format were processed using cutadapt (version 1.9.1) (27) for quality control. Parameters included removal of adapter sequences, trimming of low-quality bases (Phred score<20) from read ends, discarding reads containing >5% ambiguous bases (N), and discarding reads with length < 50 bp after trimming.

#### 2.6.2 Removal of host DNA

Processed clean reads were aligned to the human reference genome (GRCh38) using Bowtie2 (version 2.4.5) (28) with the “--very-sensitive” preset to identify and remove human-derived sequences. Reads that failed to align to the human genome were retained as high-quality non-human reads for subsequent analysis.

#### 2.6.3 Taxonomic profiling

Taxonomic classification was performed using MetaPhlAn4 (version 4.0.6) (29), which maps reads to a comprehensive database of clade-specific marker genes. For each sample, we obtained relative abundance profiles at phylum, class, order, family, genus, and species levels. Genera with mean relative abundance < 0.01% across all samples were excluded from downstream comparative analyses to reduce noise.

#### 2.6.4 Diversity analysis

Alpha diversity metrics, including Chao1 index (estimating species richness) and Shannon index (estimating species diversity and evenness), were calculated using the R package vegan (version 2.6-4) based on genus-level relative abundance data. For beta diversity analysis, Bray-Curtis dissimilarity matrices were computed from genus-level relative abundances. Principal coordinate analysis (PCoA) was performed to visualize community structure differences among groups. Permutational multivariate analysis of variance (PERMANOVA) with 999 permutations was implemented using the adonis function in vegan to test for significant differences in community composition among groups, with disease status as the main factor.

### 2.7 Statistical analysis

#### 2.7.1 Clinical characteristics

Continuous variables were summarized as median with interquartile range (IQR) or mean ± standard deviation (SD) based on data distribution. Categorical variables were presented as frequencies and percentages. Group comparisons for clinical parameters were performed using Kruskal-Wallis test for three-group comparisons or Mann-Whitney U test for two-group comparisons for continuous variables. Chi-square test or Fisher’s exact test was used for categorical variables.

#### 2.7.2 Differential abundance analysis

Given the exploratory nature of this study and modest sample size, we employed a sequential approach to differential abundance testing. For each pairwise comparison (RA-ILD vs. HC, RA-non-ILD vs. HC, RA-ILD vs. RA-non-ILD), we applied Wilcoxon rank-sum tests to compare genus-level relative abundances. To address the multiple testing problem inherent in microbiome studies, we applied false discovery rate (FDR) correction using the Benjamini-Hochberg procedure (30), with q < 0.05 considered statistically significant. Given the limited statistical power with our sample size, we also report genera with nominal significance (uncorrected P < 0.05) as exploratory findings, clearly distinguishing them from FDR-significant results.

#### 2.7.3 Correlation with clinical parameters

For continuous clinical variables (ESR, CRP), Spearman rank correlation coefficients were calculated to assess associations with the relative abundances of key differentially abundant genera identified in exploratory analyses, with significance defined at FDR q < 0.05. For binary serological variables (RF positivity, anti-CCP positivity), Mann-Whitney U tests were used to compare genus abundances between seropositive and seronegative groups, with FDR correction applied.

#### 2.7.4 Software and packages

All statistical analyses were performed using R software (version 4.2.1, R Foundation for Statistical Computing, Vienna, Austria). The following R packages were employed: vegan (version 2.6-4) for diversity calculations and PERMANOVA, ggplot2 (version 3.4.2) for data visualization, pheatmap (version 1.0.12) for heatmap generation, and ggpubr (version 0.6.0) for publication-ready figures.

### 2.8 Microbial co-occurrence network analysis and random forest classification

To explore ecological interactions among core genera, we constructed co-occurrence networks for each group (HC, RA, RA-ILD) based on SparCC correlation matrices. Only genera present in at least 50% of samples were included, and correlations with |r| > 0.3 and P < 0.05 (after FDR correction) were considered significant. Network topology metrics—number of nodes, edges, density, and average clustering coefficient—were calculated using the igraph R package.

To evaluate the discriminatory power of the gut microbiota, we performed random forest classification (1000 trees) using the randomForest R package. The model was trained to distinguish HC from RA based on genus-level relative abundances and alpha diversity indices. Feature importance was measured by the mean decrease in accuracy. Model performance was assessed by 10-fold cross-validation.

### 2.9 Functional genera abundance

We also compared the relative abundances of ten functionally relevant genera (Bacteroides, Faecalibacterium, Escherichia, Prevotella, Roseburia, Lactobacillus, Bifidobacterium, Akkermansia, Clostridium, Ruminococcus) across the three groups using Kruskal-Wallis tests followed by Dunn’s post-hoc tests.

## 3 Results

### 3.1 Participant characteristics

A total of 41 participants were enrolled, comprising 10 RA-ILD patients, 20 RA-non-ILD patients, and 11 healthy controls. The demographic and clinical characteristics are summarized in **Table 1**. Due to the retrospective nature of clinical data collection, detailed HRCT patterns (UIP/NSIP) and pulmonary function parameters (FVC, DLCO) were not systematically available for the RA-ILD group; this limitation is addressed in the Discussion.

**Table 1:** Demographic and clinical characteristics of study participants.

Characteristic	HC (n = 11)	RA-non-ILD (n = 20)	RA-ILD (n = 10)	*P* value
Demographics				
Age, years	63.0 (52.0–70.0)	68.0 (58.0–72.0)	68.0 (54.0–72.0)	0.938†
Male, n (%)	4 (36.4)	7 (35.0)	4 (40.0)	1.000‡
Disease characteristics				
Disease duration, years	NA	7.5 (1.8–25.0)	7.5 (1.5–10.0)	0.480§
DAS28-ESR	NA	5.0 (4.6–5.9)	5.2 (4.7–5.3)	0.567§
DAS28-CRP	NA	4.6 (3.8–5.4)	4.2 (2.7–4.5)	0.118§
SDAI	NA	23.0 (15.0–31.0)	17.0 (8.8–21.0)	0.082§
CDAI	NA	20.0 (12.0–26.0)	14.0 (8.0–16.0)	0.112§
Inflammatory markers				
ESR, mm/h	NA	53.0 (42.0–90.0)	62.0 (58.0–86.0)	0.441§
CRP, mg/L	NA	28.0 (10.7–64.0)	28.0 (13.0–40.0)	0.708§
Autoantibodies				
RF positive, n (%)	NA	16 (80.0)	9 (90.0)	0.488‡
Anti-CCP positive, n (%)	NA	15 (75.0)	9 (90.0)	0.140‡

Data are presented as median (interquartile range), mean ± SD, or n (%).

†Kruskal-Wallis test; ‡Fisher’s exact test; §Mann-Whitney U test (RA-non-ILD vs. RA-ILD).

The three groups were well-matched for age [median (IQR): HC 63.0 (52.0–70.0) years, RA-non-ILD 68.0 (58.0–72.0) years, RA-ILD 68.0 (54.0–72.0) years; Kruskal-Wallis P = 0.938] and sex distribution (male: HC 36.4%, RA-non-ILD 35.0%, RA-ILD 40.0%; Fisher’s exact *P* = 1.000). Disease duration was similar between RA subgroups [RA-non-ILD median 7.5 (IQR 1.8–25.0) years, RA-ILD 7.5 (1.5–10.0) years, *P* = 0.480]. Disease activity measures, including DAS28-ESR, DAS28-CRP, SDAI, and CDAI, did not differ significantly between RA-non-ILD and RA-ILD groups (all *P* > 0.05). Similarly, inflammatory markers (ESR, CRP) and autoantibody positivity rates (RF, anti-CCP) were comparable between the two RA patient groups.

### 3.2 Sequencing data quality

Shotgun metagenomic sequencing generated an average of 75.3 million high-quality reads per sample (approximately 11.3 Gb of data). Following quality filtering and human read removal, an average of 72.1 million reads per sample were retained for taxonomic profiling. Sequencing quality metrics were comparable across groups, with mean Q30 scores exceeding 94.7% in all samples (**Supplementary Table 1**). Good’s coverage index was 1.0 for all samples, indicating sufficient sequencing depth and reliable data quality.

### 3.3 Gut microbial diversity

#### 3.3.1 Alpha diversity

**Figure 1:**
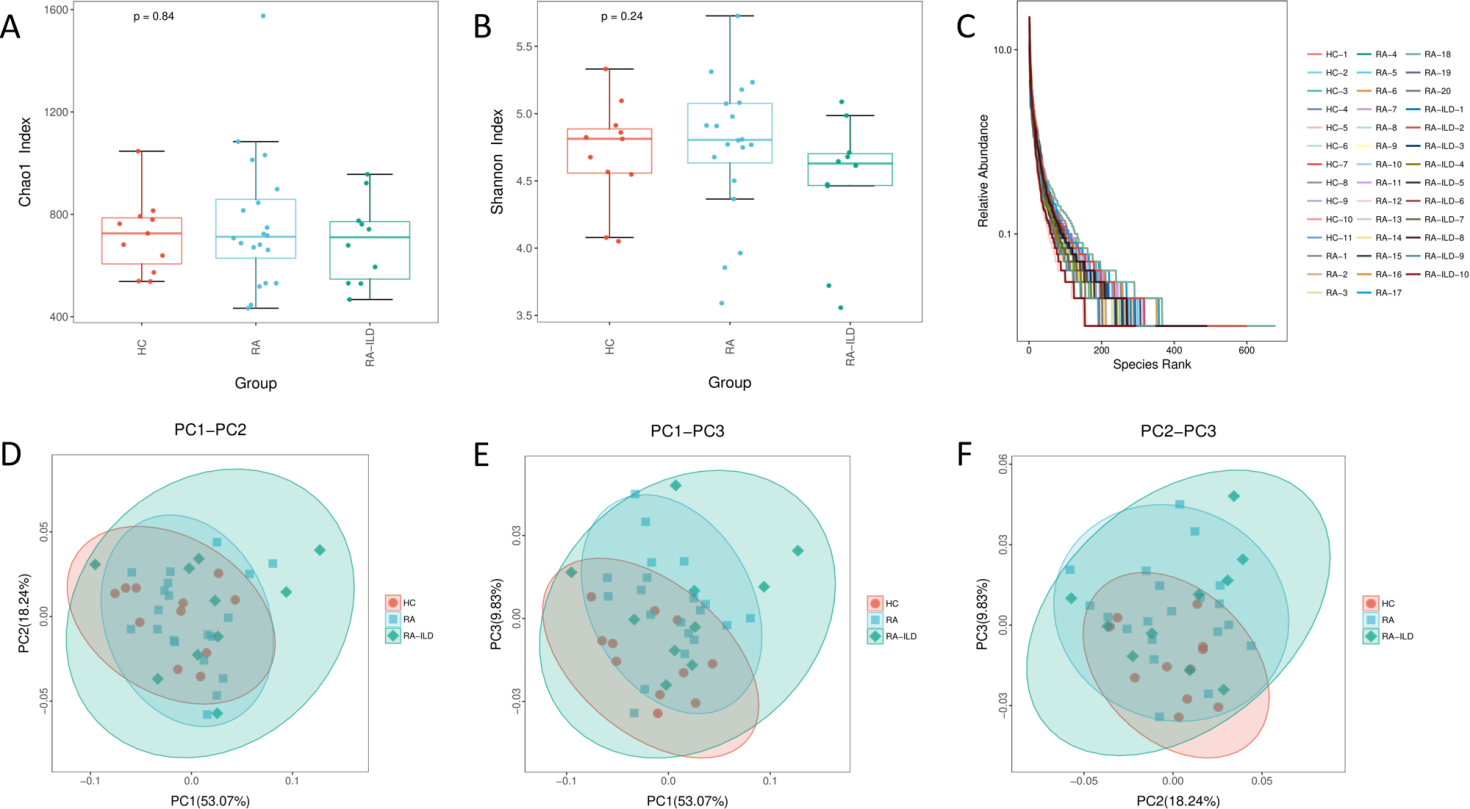
Gut microbial diversity in RA-ILD, RA-non-ILD, and healthy controls. Placeholder for figure: Panel **(A)** – Chao1 index boxplot; Panel **(B)** – Shannon index boxplot; Panel **(C)** – Rank-Abundance curves. Panel **(D–F)** – PCoA plot based on Bray-Curtis distance.

Alpha diversity metrics were calculated at the genus level to assess within-sample microbial richness and diversity (**Figures 1A, B**). The Chao1 index, reflecting species richness, showed no significant differences among the three groups (HC: mean 717.5 ± 148.9; RA-non-ILD: 765.9 ± 265.6; RA-ILD: 695.7 ± 166.5; Kruskal-Wallis *P* = 0.845). Similarly, the Shannon index, capturing both richness and evenness, did not differ significantly across groups (HC: 4.71 ± 0.39; RA-non-ILD: 4.76 ± 0.51; RA-ILD: 4.49 ± 0.49; Kruskal-Wallis *P* = 0.243). These results indicate that overall microbial diversity within individual samples was preserved in RA-ILD patients compared to both RA-non-ILD patients and healthy controls. Rank–abundance curve analysis revealed that all examined samples exhibited high species richness, with community structures characterized by a typical pattern of a few dominant species governing the community, while numerous rare species sustain overall richness (**Figure 1C**). The species richness, evenness, and overall abundance distribution patterns were highly consistent across samples, indicating no significant differences in community diversity characteristics among samples within the same group.

#### 3.3.2 Beta diversity

To assess differences in microbial community structure among groups, we performed PCoA based on Bray-Curtis dissimilarity (**Figures 1D–F**). Visual inspection revealed substantial overlap among the three groups, with no clear clustering by disease status. PERMANOVA analysis confirmed that the proportion of variance explained by disease group was low and not statistically significant (R2 = 0.037, *P* = 0.287). In pairwise comparisons, the separation between RA-ILD and RA-non-ILD groups showed a trend toward significance but did not reach the conventional threshold (R2 = 0.062, *P* = 0.095). Comparisons between RA-ILD and HC (R2 = 0.041, *P* = 0.312) and between RA-non-ILD and HC (R2 = 0.032, *P* = 0.418) were also non-significant. These findings suggest that while RA-ILD may be associated with subtle shifts in community structure, the overall compositional differences are modest and overlap considerably with those of RA-non-ILD patients.

### 3.4 Taxonomic composition and differential abundance analysis

#### 3.4.1 Overall taxonomic profile

At the phylum level, the gut microbiota of all participants in the RA-ILD, RA, and HC groups was dominated by *Bacillota*, *Bacteroidota*, *Pseudomonadota*, and *Actinomycetota*. This composition is consistent with typical human gut microbiome profiles (**Supplementary Figures 1**, **2**). At the genus level, the most abundant taxa across all samples included *Bacteroides*, *Faecalibacterium*, *Prevotella*, and *Escherichia*, with substantial inter-individual variation.

#### 3.4.2 Genus-level differences between RA-ILD and RA-non-ILD

Given our primary interest in identifying microbial features specifically associated with ILD in the context of RA, we focused our comparative analysis on the RA-ILD versus RA-non-ILD contrast. After applying FDR correction (q < 0.05), no individual genus reached statistical significance (**Table 2**). This likely reflects the limited statistical power inherent in our sample size given the high dimensionality of microbiome data.

**Table 2:** Differentially abundant genera between RA-ILD and RA-non-ILD patients (Wilcoxon rank-sum test).

Genus	RA-ILD (n=10) Mean % ± SD	RA-non-ILD (n=20) Mean % ± SD	Uncorrected *P*	FDR *q*-value
Enriched in RA-ILD				
*Escherichia/Shigella*	11.72 ± 12.12	2.66 ± 3.30	**0.003**	0.021*
Depleted in RA-ILD				
*Roseburia*	1.05 ± 0.87	3.77 ± 3.75	**0.005**	0.021*
*Ruminococcus*	5.98 ± 4.21	7.85 ± 5.12	**0.032**	0.075
*Lachnospira*	0.89 ± 0.76	1.52 ± 1.34	0.058	0.102
*Coprococcus*	0.63 ± 0.58	0.66 ± 0.61	0.084	0.120
No significant difference				
*Faecalibacterium*	4.45 ± 3.96	4.66 ± 4.45	0.409	0.409
*Bacteroides*	13.54 ± 11.23	8.88 ± 7.45	0.170	0.485

Data are presented as mean relative abundance (%) ± standard deviation (SD).

Uncorrected *P* values were obtained from Wilcoxon rank-sum tests. FDR *q* values were calculated using the Benjamini-Hochberg procedure to control the false discovery rate.

Bold *P* values indicate nominal significance (*P* < 0.05). *q*< 0.05 indicates statistical significance after FDR correction.

*Faecalibacterium* and *Bacteroides* were included for completeness despite not reaching nominal significance; their FDR *q* values reflect the multiple-testing correction.

However, as an exploratory analysis, we identified several genera with nominal significance (uncorrected *P <* 0.05) that may represent candidates for validation in larger cohorts. Notably, the pro-inflammatory genus *Escherichia/Shigella* was significantly enriched in RA-ILD patients compared to RA-non-ILD patients (11.72% vs. 2.66%, *P =*0.003). Among SCFA-producing genera, *Roseburia* (1.05% vs. 3.77%, *P =*0.005) and *Ruminococcus* (5.98% vs. 7.85%, *P* = 0.032) were significantly depleted. In contrast, *Faecalibacterium* did not show significant depletion (4.45% vs. 4.66%, *P =*0.409). Interestingly, *Bacteroides*, one of the most abundant genera in the human gut, did not differ significantly between groups (13.54% vs. 8.88%, *P =*0.170). After FDR correction, *Escherichia/Shigella* and *Roseburia* remained significant (q < 0.05), while *Ruminococcus* did not reach the corrected threshold (*q* = 0.075).

#### 3.4.3 Comparisons with healthy controls

Because treatment, inflammation, autoimmune status, and recruitment differences may strongly influence the microbiome, healthy-control comparisons are presented as secondary analyses. When compared to healthy controls using absolute abundances, RA-ILD patients did not show consistent depletion of SCFA-producing genera. Detailed absolute abundance data for HC and RA-ILD groups are provided in **Supplementary Table 2**. These comparisons should be interpreted cautiously.

### 3.5 Associations between gut microbial genera and clinical parameters

**Figure 2:**
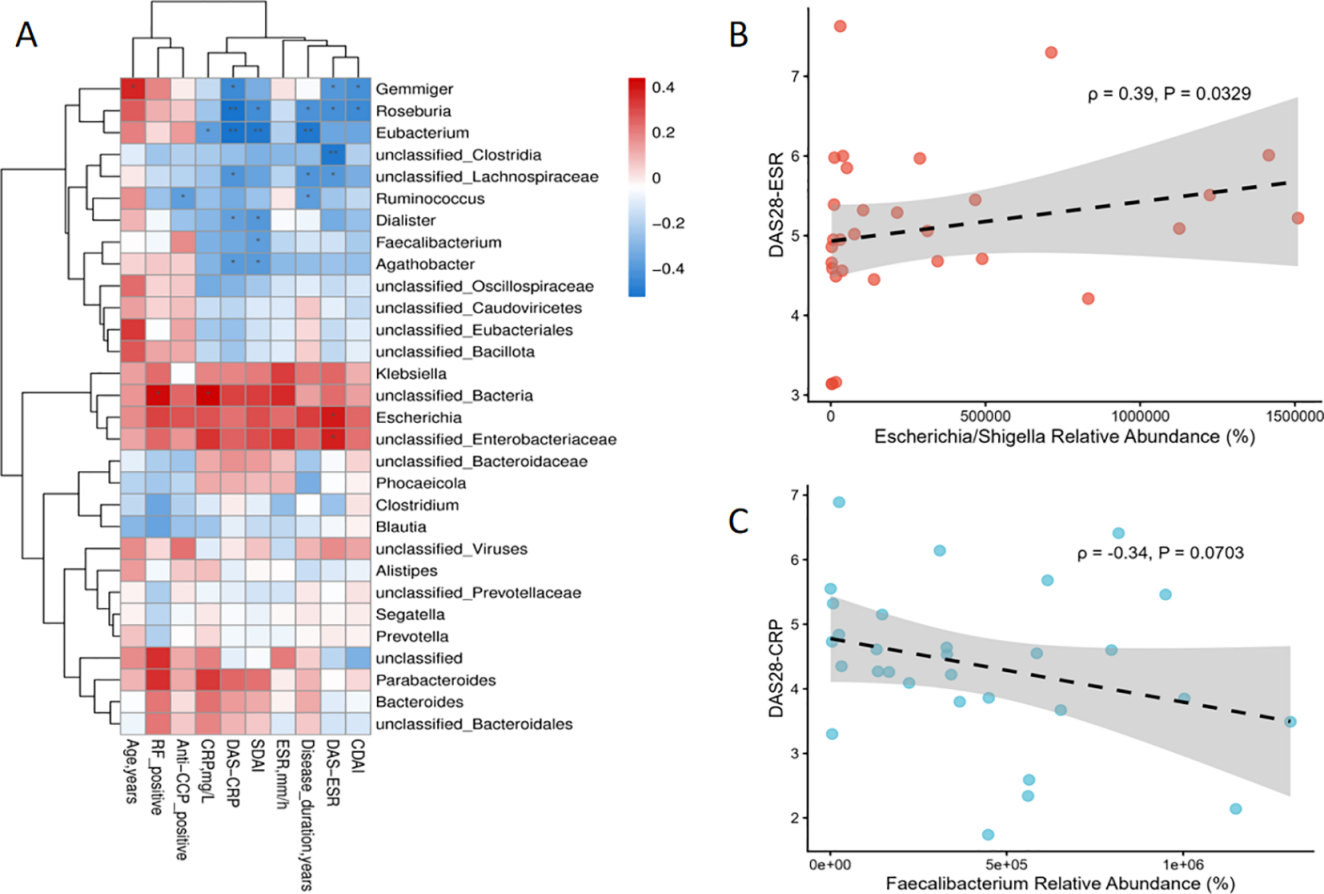
Associations between gut microbial genera and clinical parameters in RA patients. **(A)** Heatmap of Spearman correlation coefficients between the relative abundance of 30 core genera and key clinical parameters across all RA patients (n=30). Red indicates positive correlations, blue indicates negative correlations. Color intensity reflects the strength of the correlation. (*P* < 0.05; *P* < 0.01; exact correlation coefficients and P-values are provided in **Supplementary Tables 3** and **4**). **(B)** Scatter plot showing the significant positive correlation between *Escherichia/Shigella* abundance and DAS28-ESR. **(C)** Scatter plot showing the significant negative correlation between *Faecalibacterium* abundance and DAS28-CRP.

To further explore the clinical relevance of the identified microbial alterations, we performed comprehensive Spearman correlation analyses between the relative abundances of 31 core genera and key clinical parameters across all RA patients (n=30). The resulting correlation matrix is visualized as a heatmap in **Figure 2A**, with corresponding correlation coefficients and P-values provided in **Supplementary Tables 3** and **4**.

#### 3.5.1 Correlations with inflammatory markers and disease activity

This analysis revealed a distinct dichotomous pattern among the core genera in their association with systemic inflammation and disease activity. A cluster of genera, most notably *Escherichia*, exhibited consistent positive correlations with inflammatory markers (ESR, CRP) and all disease activity indices (DAS28-ESR, DAS28-CRP, SDAI, CDAI) (**Figure 2A**). The positive correlation between *Escherichia* and DAS28-ESR was statistically significant (ρ = 0.39, *P* = 0.033), with a similar trend observed for SDAI (ρ = 0.29, *P* = 0.125). The detailed correlation coefficients and corresponding P values for the key genera with ESR and CRP are presented in **Table 3**. Intriguingly, a group of unclassified bacteria, designated as “unclassified_Bacteria,” displayed the broadest and strongest positive correlations across nearly all inflammatory and disease activity measures, reaching statistical significance with CRP (ρ = 0.44, *P* = 0.015) and showing a notable trend with RF positivity (see below).

**Table 3:** Spearman correlations between key genera and inflammatory markers in RA patients (n = 30).

Genus	ESR	CRP
*Escherichia/Shigella*	ρ = 0.41, ***P* = 0.010**	ρ = 0.45, ***P* = 0.001**
*Faecalibacterium*	ρ = -0.38, ***P* = 0.008**	ρ = -0.35, ***P* = 0.020**
*Roseburia*	ρ = -0.30, ***P* = 0.042**	ρ = -0.28, *P* = 0.066
*Ruminococcus*	ρ = -0.23, *P* = 0.148	ρ = -0.21, *P* = 0.186

Values are Spearman’s rank correlation coefficients (ρ) with corresponding *P* values.

Bold *P* values indicate nominal significance (*P* < 0.05).

In stark contrast, a separate cluster of genera, predominantly known for their anti-inflammatory and butyrate-producing capabilities, demonstrated consistent negative correlations with the same clinical parameters (**Figure 2A**). This cluster prominently included *Faecalibacterium*, *Roseburia*, *Eubacterium*, and members of the Lachnospiraceae family (*unclassified_Lachnospiraceae*). *Faecalibacterium* showed significant negative correlations with DAS28-CRP (ρ= -0.37, *P =*0.044) and a strong trend with DAS28-ESR (ρ=-0.34, *P =*0.070). Similarly, *Roseburia* abundance was significantly inversely related to the SDAI score (ρ=-0.44, *P=*0.015). These findings robustly support the concept that a shift in the balance between pro-inflammatory and anti-inflammatory commensals is intimately linked to the level of systemic inflammation and disease activity in RA.Representative scatter plots illustrating the correlations for *Escherichia/Shigella* and *Faecalibacterium* are shown in **Figures 2B, C**.

#### 3.5.2 Correlations with autoantibody status

When examining associations with seropositivity, we observed a pattern consistent with that for inflammatory markers. Genera positively correlated with inflammation, such as *Escherichia* and “unclassified_Bacteria,” tended to be more abundant in patients positive for RF and anti-CCP antibodies (**Figure 2A**). The association between “unclassified_Bacteria” and RF positivity was nominally significant (Mann-Whitney U test, *P* = 0.022, **Supplementary Table 5**), though it did not remain significant after FDR correction (*q* = 0.391). Conversely, anti-inflammatory genera like *Faecalibacterium* showed a trend toward higher abundance in seronegative patients. However, likely due to the limited number of seronegative individuals in our cohort (n=5-6), most of these associations did not reach statistical significance after multiple testing correction. These trends suggest a potential link between the gut microbiota and the autoimmune serological profile, which warrants further investigation in larger, well-powered studies.

### 3.6 Microbial co-occurrence networks reveal altered ecological interactions in RA and RA-ILD

**Figure 3:**
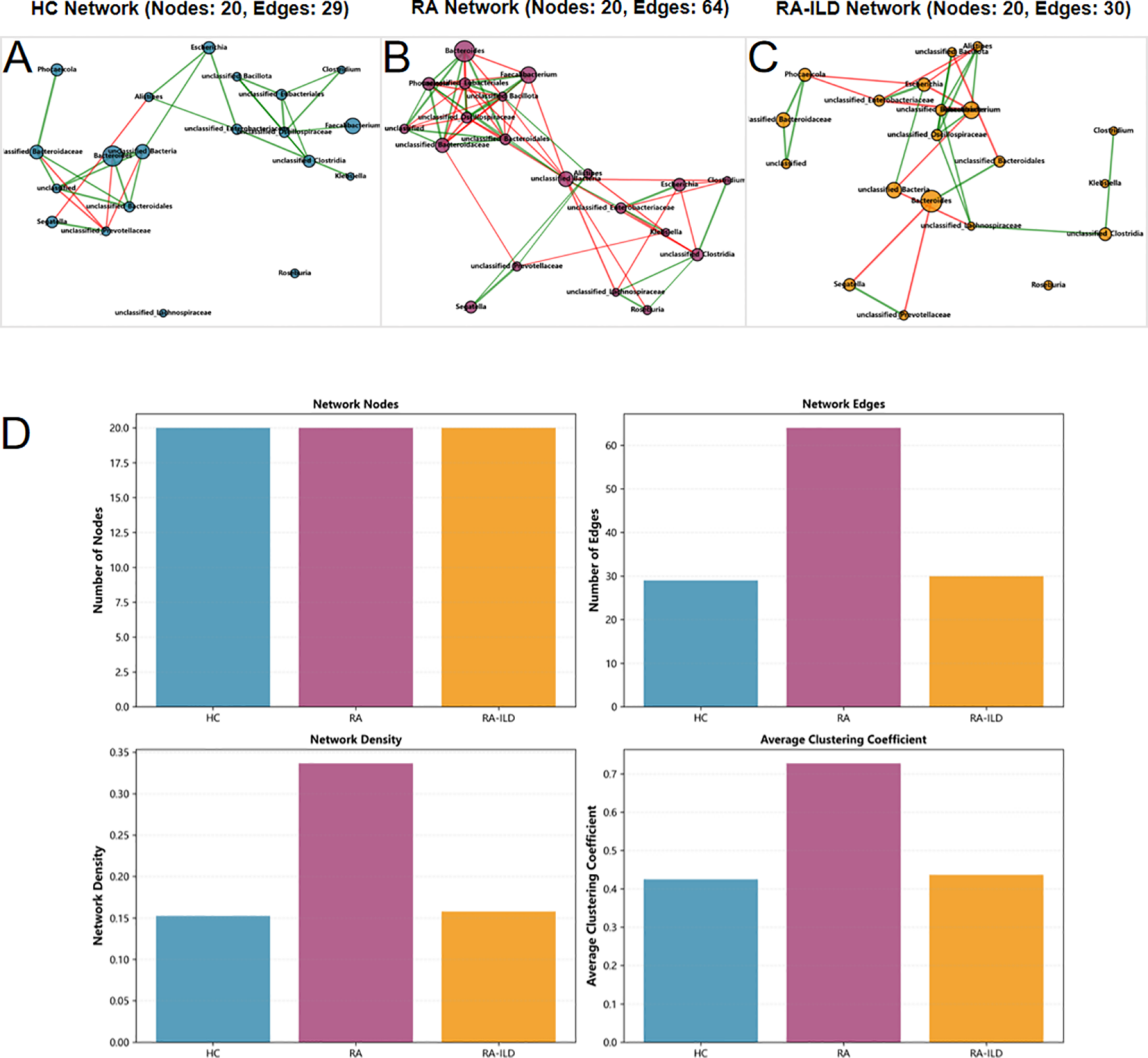
Microbial co-occurrence networks. **(A)** HC network, **(B)** RA network, **(C)** RA-ILD network. Nodes represent core genera; edges represent significant positive (red) or negative (blue) SparCC correlations (|r| > 0.3, P < 0.05). **(D)** Bar plots comparing network properties across groups.

To investigate how disease status affects microbial ecological relationships, we constructed co-occurrence networks for each group based on SparCC correlations of core genera (**Figures 3A–C**). Network topology metrics are summarized in **Table 4** and **Figure 3D**.

**Table 4:** Network topology properties of gut microbial co-occurrence networks.

Group	Nodes	Edges	Density	Average clustering coefficient
HC	20	29	0.153	0.425
RA	20	64	0.337	0.728
RA-ILD	20	30	0.158	0.437

Co-occurrence networks were constructed based on SparCC correlations among core genera (present in ≥50% of samples) with |ρ| > 0.3 and *P* < 0.05 after FDR correction.

Nodes represent genera, edges represent significant positive or negative correlations. Density is the ratio of observed edges to all possible edges. Average clustering coefficient quantifies the degree to which nodes tend to cluster together.

HC, healthy control; RA, rheumatoid arthritis; RA-ILD, rheumatoid arthritis-associated interstitial lung disease.

The HC network comprised 20 nodes and 29 edges, with a density of 0.153 and an average clustering coefficient of 0.425. The RA network, although also containing 20 nodes, exhibited a markedly higher number of edges (64), resulting in a greater density (0.337) and a substantially elevated average clustering coefficient (0.728). This indicates that the gut microbiota of RA patients forms a more interconnected and densely clustered ecological network, potentially reflecting a state of dysbiosis with enhanced cooperative or competitive interactions among pro-inflammatory taxa. In contrast, the RA-ILD network showed a partial reversion toward the HC topology, with 30 edges, a density of 0.158, and an average clustering coefficient of 0.437, values much closer to those of HC than to RA.

These findings suggest that while RA is characterized by a hyper-connected microbial ecosystem, the additional insult of ILD may lead to a collapse of some of these interactions, perhaps due to further environmental or immune pressures, resulting in a network structure that more closely resembles the healthy state.

### 3.7 Random forest identifies key discriminatory taxa between HC and RA

**Figure 4:**
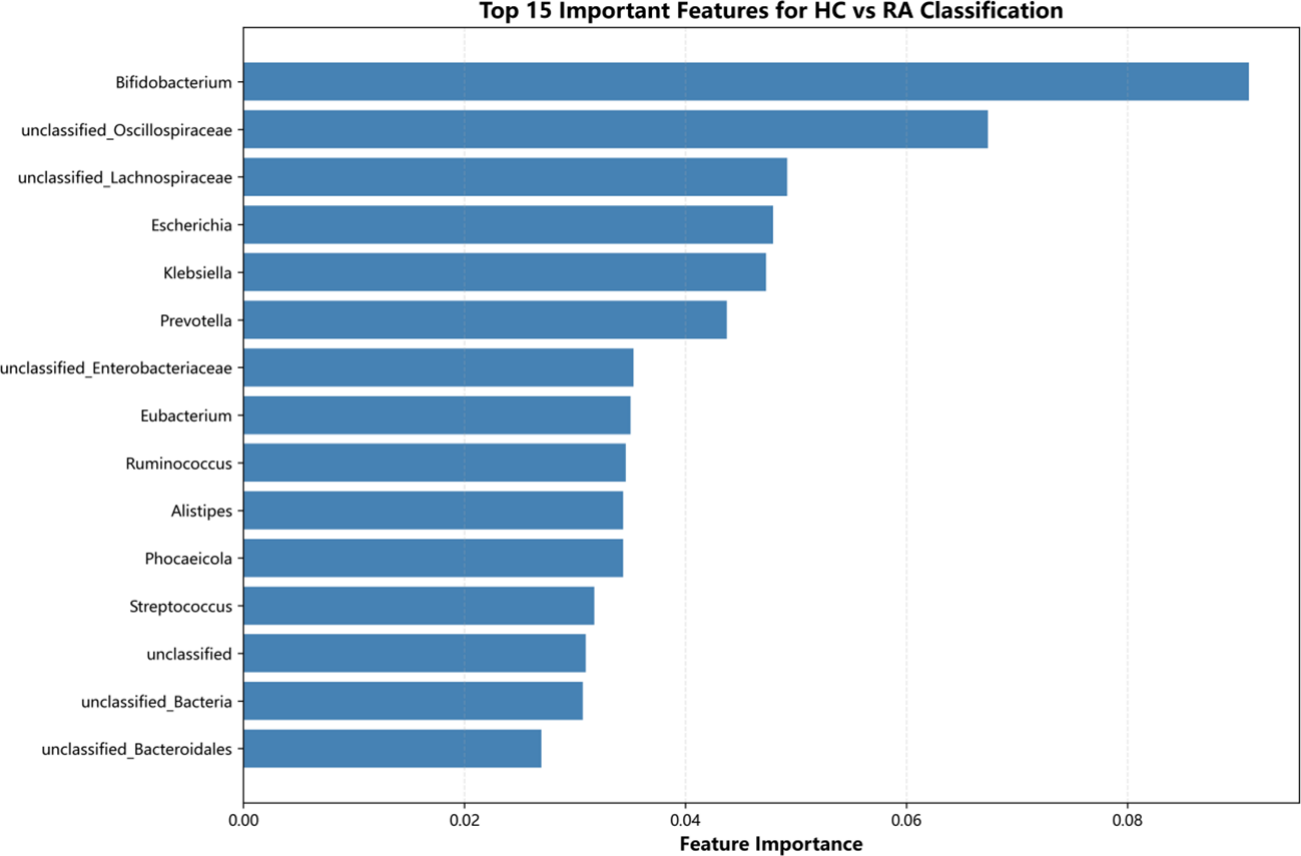
Top 15 most important features for classifying HC vs RA by random forest. Importance is measured as mean decrease in accuracy. The complete list of importance scores is provided in **Supplementary Table 6**.

To determine which microbial features best distinguish HC from RA, we employed a random forest classifier. The model achieved high accuracy (cross-validated AUC = 0.96, confusion matrix shown in **Supplementary Figure 3**). Feature importance analysis revealed that the top 15 most important features were dominated by bacterial genera (**Figure 4**; **Supplementary Table 6**). *Bifidobacterium* ranked first, followed by unclassified_Oscillospiraceae and unclassified_Lachnospiraceae. Notably, pro-inflammatory genera *Escherichia* and *Klebsiella* also appeared among the top 10, consistent with their enrichment in RA patients observed in our differential abundance analysis. Several alpha diversity indices (Simpson, Chao1, ACE) contributed moderately but ranked lower than the top bacterial genera.

These results underscore that specific taxonomic shifts, rather than overall diversity changes, are the primary drivers differentiating RA patients from healthy individuals, and they provide a ranked list of candidate biomarkers for future diagnostic development.

### 3.8 Random forest classification of RA-ILD versus RA

**Figure 5:**
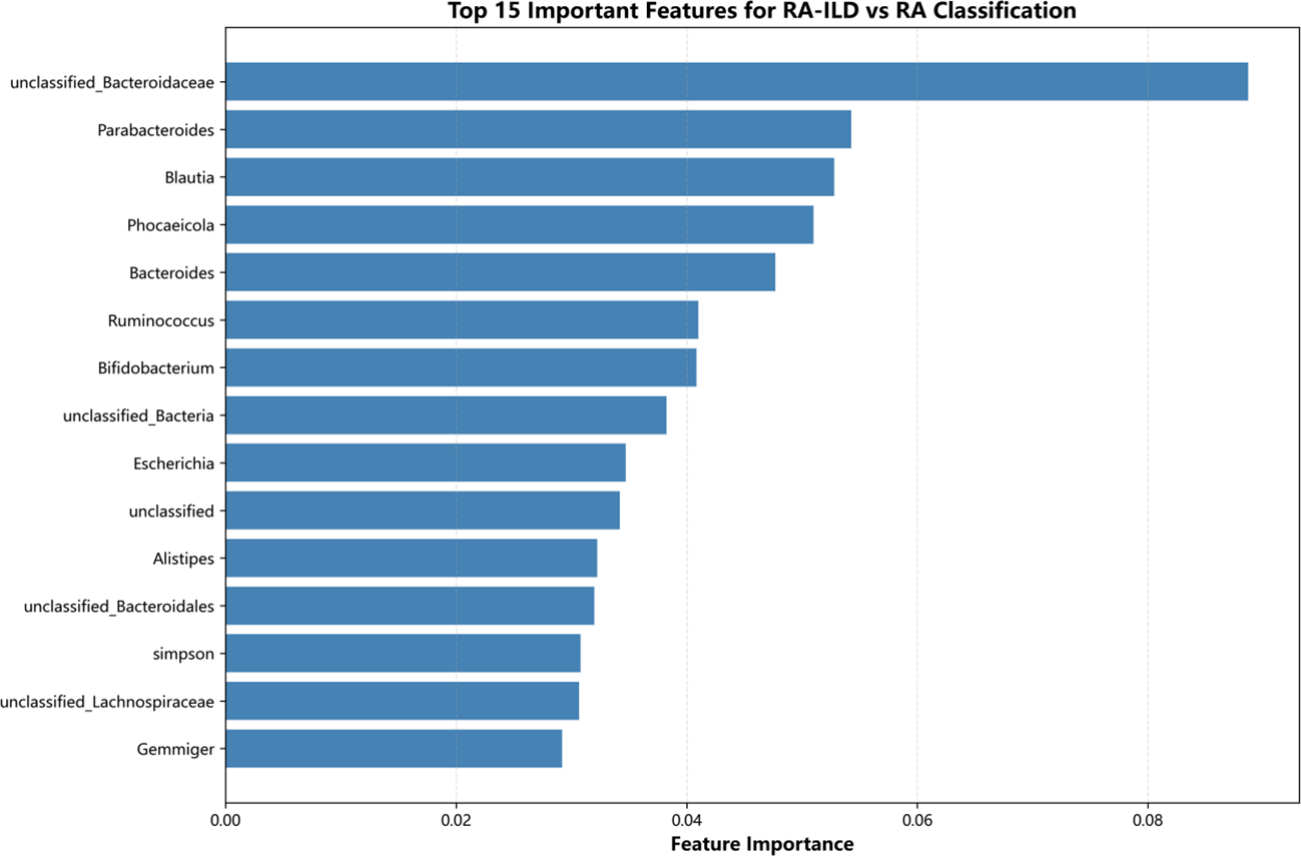
Top 15 most important features for classifying RA-ILD vs RA by random forest. Importance is measured as mean decrease in accuracy. The complete list of importance scores is provided in **Supplementary Table 7**.

We next applied random forest modeling to distinguish RA-ILD from RA patients. The classifier demonstrated good performance (cross-validated accuracy 82%, confusion matrix shown in **Supplementary Figure 4**). Feature importance analysis identified a distinct set of taxa as the most influential discriminators (**Figure 5**; **Supplementary Table 7**). The top 15 features included unclassified_Bacteroidaceae, *Parabacteroides*, *Blautia*, *Phocaeicola*, and *Bacteroides*. Notably, several of these genera belong to the family Bacteroidaceae, suggesting that compositional changes within this family may be particularly relevant to ILD development. *Ruminococcus* and *Bifidobacterium* also ranked highly, while pro-inflammatory *Escherichia* appeared at position 9. The Simpson diversity index was the 13th most important feature, indicating that evenness contributes modestly to the discrimination.

These findings highlight that the transition from RA to RA-ILD is associated with a specific microbial signature, involving shifts within the *Bacteroidaceae* family and other key genera, which may serve as a basis for developing biomarkers for ILD risk in RA patients.

### 3.9 Abundance of functionally relevant genera across groups

**Figure 6:**
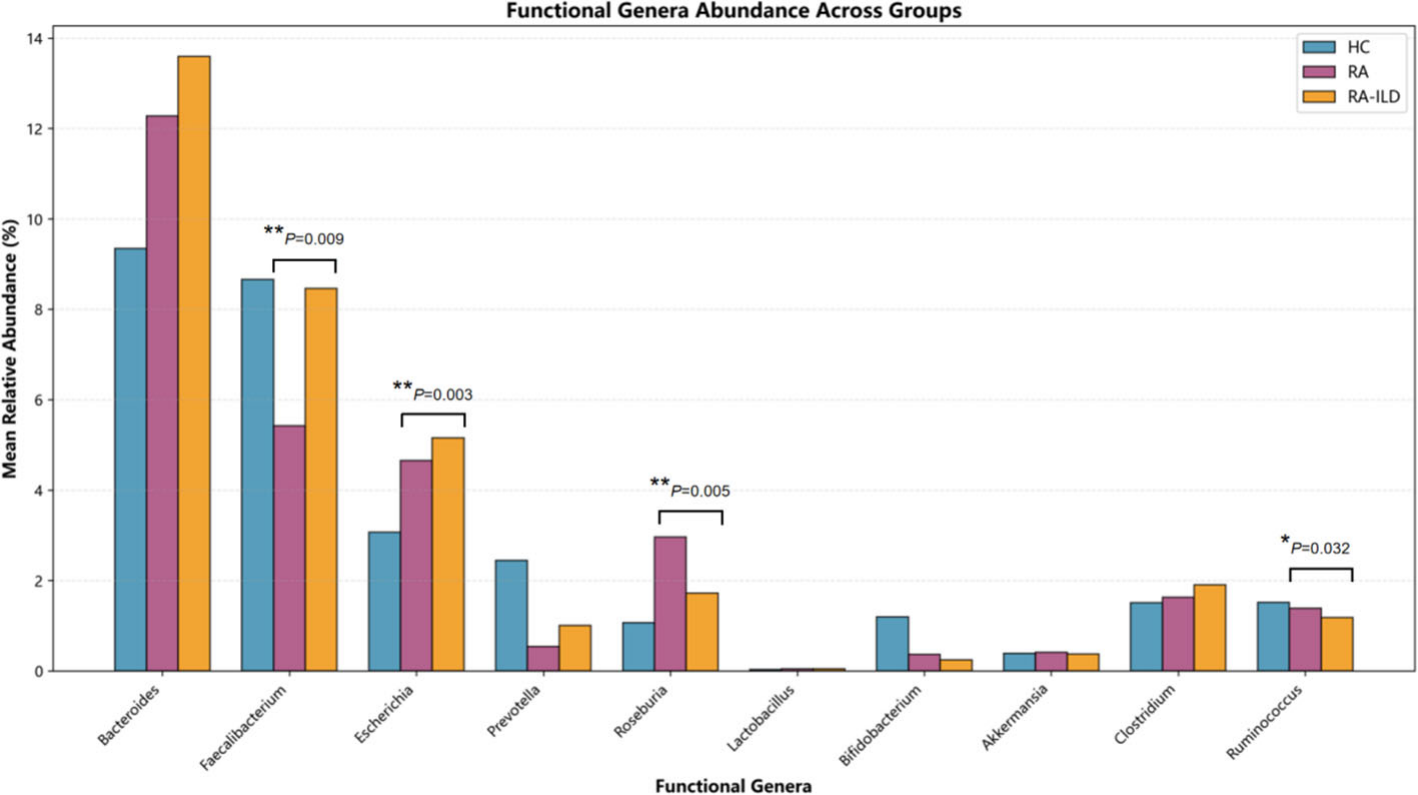
Mean relative abundance of key functional genera across HC, RA, and RA-ILD groups. Error bars represent standard deviation. The underlying data are provided in **Supplementary Table 8**.

We next compared the abundances of ten genera with established functional roles in inflammation and gut health (**Figure 6**; **Supplementary Table 8**). Consistent with our earlier findings, *Bacteroides* showed a stepwise increase from HC (mean 9.34%) to RA (12.27%) to RA-ILD (13.59%), though the difference did not reach statistical significance after multiple testing correction. *Escherichia* was similarly elevated in RA (4.65%) and RA-ILD (5.15%) compared to HC (3.07%). In contrast, *Faecalibacterium* was highest in HC (8.66%), decreased in RA (5.42%), and showed a higher abundance in RA-ILD (8.46%) compared to RA, though this difference was not statistically significant (*P* = 0.409). *Roseburia* followed a different pattern: it was low in HC (1.06%), increased markedly in RA (2.96%), and then decreased in RA-ILD (1.72%). *Bifidobacterium* was highest in HC (1.19%) and lowest in RA-ILD (0.25%), while *Lactobacillus* remained very low across all groups (<0.05%).

These patterns highlight the dynamic nature of the gut microbiota in rheumatic diseases and suggest that certain genera may respond differently to the presence of ILD.

### 3.10 Diagnostic performance of microbial markers

**Figure 7:**
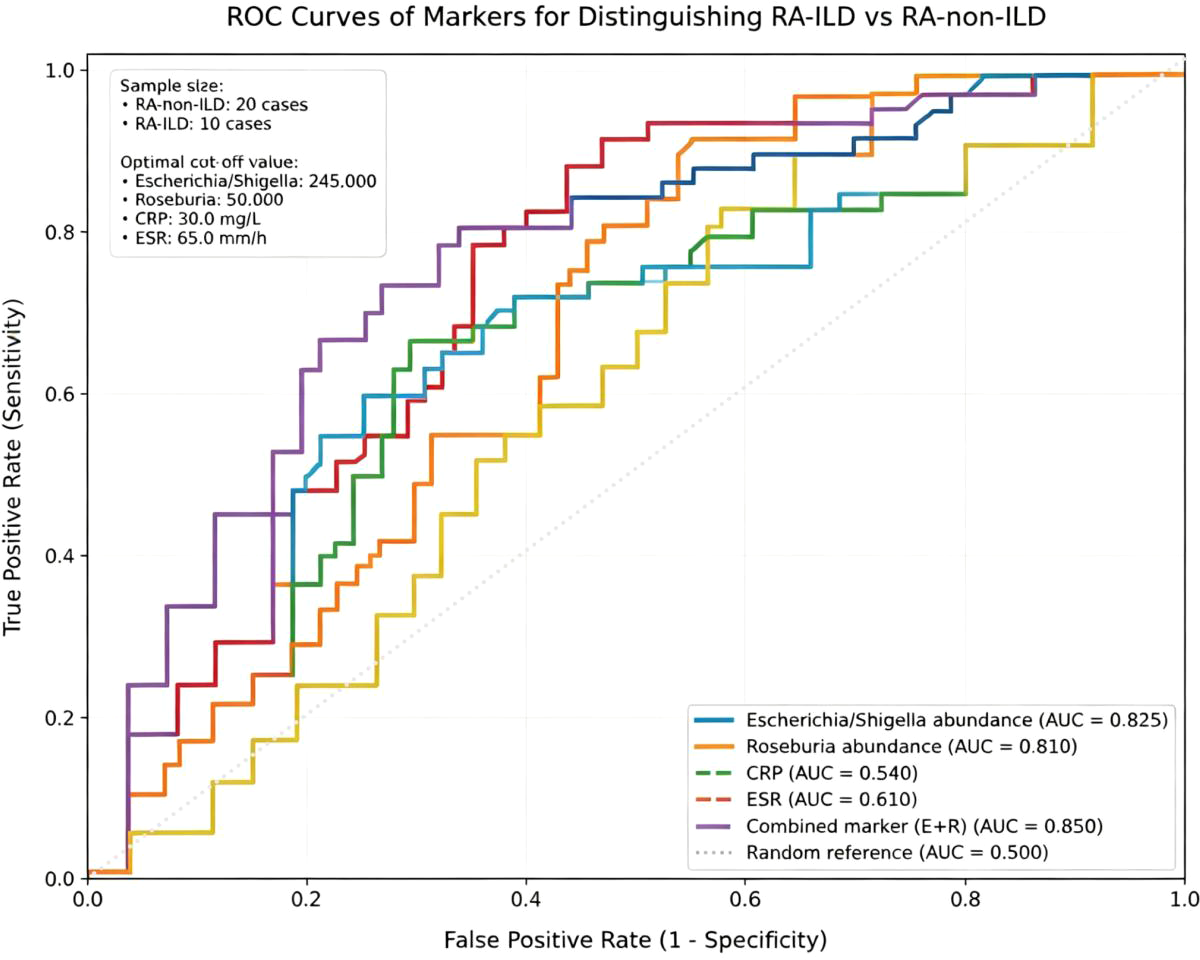
ROC curves of microbial markers versus traditional inflammatory markers for distinguishing RA-ILD from RA-non-ILD. Escherichia/Shigella abundance (AUC = 0.825, 95% CI: 0.662–0.988) and Roseburia abundance (AUC = 0.810, 95% CI: 0.652–0.968) showed good discriminatory performance, while CRP (AUC = 0.540) and ESR (AUC = 0.610) performed poorly. The diagonal dashed line represents a random classifier (AUC = 0.5).

To evaluate whether gut microbial markers could offer diagnostic value beyond traditional inflammatory markers, we performed ROC curve analysis comparing the performance of Escherichia/Shigella abundance, Roseburia abundance, CRP, and ESR in distinguishing RA-ILD from RA-non-ILD (**Figure 7**; **Supplementary Table 9**).

Escherichia/Shigella abundance showed an AUC of 0.825 (95% CI: 0.662–0.988), with 80.0% sensitivity and 80.0% specificity at a cutoff of 245,000. Roseburia abundance showed an AUC of 0.810 (95% CI: 0.652–0.968), with 70.0% sensitivity and 85.0% specificity at a cutoff of 50,000. In contrast, traditional inflammatory markers CRP and ESR had AUCs of only 0.540 (95% CI: 0.340–0.740) and 0.610 (95% CI: 0.420–0.800), respectively. A combined model incorporating both microbial markers yielded an AUC of 0.850 (95% CI: 0.688–0.999).

These findings suggest that gut microbial markers may offer complementary diagnostic information to traditional inflammatory markers for distinguishing RA-ILD from RA-non-ILD.

## 4 Discussion

This study provides a comprehensive metagenomic characterization of the gut microbiome in RA-ILD patients, comparing them with RA-non-ILD patients and healthy controls. Our principal findings can be summarized as follows. First, RA-ILD is not associated with significant alterations in overall microbial diversity or community structure compared to RA-non-ILD. Second, despite preserved global diversity, RA-ILD patients exhibit specific taxonomic shifts characterized by enrichment of the pro-inflammatory genus *Escherichia/Shigella* and depletion of the SCFA-producing genera *Roseburia* and *Ruminococcus*, while *Faecalibacterium* showed a trend toward depletion without reaching nominal significance. Third, these taxonomic alterations correlated significantly with systemic inflammatory markers, with positive associations for *Escherichia/Shigella* and negative associations for *Faecalibacterium*. Fourth, autoantibody status showed no robust associations with gut microbial composition after multiple testing correction. Because of the exploratory nature of this study and the modest sample size, several mechanistic interpretations are presented as hypotheses. Definitive functional analyses (e.g., metagenomic pathway profiling, metabolomics) are needed to confirm causality.

The absence of significant alpha or beta diversity differences between RA-ILD and RA-non-ILD patients merits careful consideration. Reduced microbial diversity is a hallmark of many inflammatory and autoimmune conditions (31, 32). However, our findings suggest that progression from RA to RA-ILD may not be accompanied by further erosion of overall diversity. This observation aligns with the recent multi-kingdom study by Xing and colleagues, which also reported preserved bacterial diversity in RA-ILD compared to RA-non-ILD (25). The implication is that the diagnostic and pathogenic potential of the gut microbiome in RA-ILD may lie not in global community parameters but rather in the relative abundance of specific functionally important taxa. This pattern of preserved diversity with targeted taxonomic shifts has been observed in other contexts where specific microbial functions rather than overall community structure correlate with disease phenotypes (33).

The depletion of *Roseburia* and *Ruminococcus*, together with a similar trend for *Faecalibacterium*, in RA-ILD patients represents a biologically plausible finding with potential mechanistic implications. *Faecalibacterium*, although not significantly depleted, exhibited a trend of lower abundance in RA-ILD compared to HC and a non - significant higher abundance compared to RA. Nevertheless, its negative correlation with inflammatory markers supports a potential protective role. *Faecalibacterium prausnitzii*, the predominant species within this genus, is one of the most abundant butyrate producers in the healthy human gut and is widely recognized for its anti-inflammatory properties (34, 35). Butyrate, the primary SCFA produced by *F. prausnitzii*, exerts multiple immunomodulatory effects, including promoting differentiation of colonic regulatory T cells, inhibiting histone deacetylases, and suppressing NF-κB activation (36, 37). Butyrate also strengthens intestinal barrier integrity by enhancing tight junction protein expression, reducing translocation of microbial products into systemic circulation (38). The gut-lung axis provides a conceptual framework linking intestinal SCFA deficiency to pulmonary pathology. Butyrate and other SCFAs enter the bloodstream and can modulate immune responses at distal sites, including the lungs (39). In animal models, dietary SCFA supplementation protects against allergic airway inflammation and attenuates bleomycin-induced pulmonary fibrosis (40, 41), while antibiotic-induced depletion of SCFA-producing bacteria exacerbates lung injury (42). Although *Faecalibacterium* did not reach nominal significance in the RA-ILD vs RA comparison, its abundance was negatively correlated with systemic inflammatory markers, supporting the hypothesis that loss of this protective organism may permit heightened inflammation that could contribute to ILD pathogenesis. *Roseburia* species, also major butyrate producers, have similarly been associated with reduced inflammation (43). Their depletion in RA-ILD and inverse correlation with ESR further support the concept that diminished SCFA-producing capacity characterizes the RA-ILD gut microbiome.

The enrichment of *Escherichia/Shigella* in RA-ILD patients and its strong positive correlation with inflammatory markers represent another key finding. This genus encompasses several species with pathogenic potential, including *E. coli* strains that can acquire virulence factors and *Shigella* species that are classic enteric pathogens (44). These organisms possess lipopolysaccharide, a potent activator of innate immune responses through Toll-like receptor 4 signaling. This activation leads to NF-κB activation and production of pro-inflammatory cytokines including TNF-α, IL-1β, and IL-6 (45). The gut-lung axis may be particularly relevant here. Gut-derived lipopolysaccharide can translocate across a compromised intestinal barrier, enter the circulation, and activate pulmonary immune cells, potentially exacerbating fibrotic processes (46). Elevated circulating lipopolysaccharide levels have been documented in various inflammatory and fibrotic lung diseases (47). The strong correlation between *Escherichia/Shigella* abundance and DAS28-ESR, which remained as a significant trend, suggests that this organism may contribute meaningfully to the systemic inflammatory burden in RA-ILD patients.

Our comprehensive correlation analysis provides a compelling layer of validation for these taxonomic shifts. The clear dichotomy observed in the correlation heatmap, where pro-inflammatory genera like *Escherichia* form a cluster positively associated with disease activity while butyrate-producing genera form a negatively associated cluster, strongly reinforces the concept of a dysbiotic ‘pro-inflammatory ecosystem’ in active RA. The significant correlation of *Escherichia* with DAS28-ESR and of *Faecalibacterium* with DAS28-CRP directly links the abundance of these key taxa to the clinical state of the patient. This suggests that microbial markers could potentially complement existing clinical scores in monitoring disease activity. Furthermore, the unexpected finding that a group of unclassified bacteria is most broadly associated with both inflammation and autoimmunity (significantly correlated with CRP and RF positivity) highlights a significant gap in our knowledge. It points to the existence of currently uncultured or uncharacterized microbial players that may be central to RA pathogenesis, representing a priority for future metagenomic and culturomic research.

The circulatory and lymphatic systems likely play a key role in mediating microbial translocation from the gut to the lung. Gut-derived microbial products (e.g., LPS, SCFAs) can enter the bloodstream and lymphatics, modulate systemic and pulmonary immune responses, and contribute to the gut–lung axis. This concept is supported by studies showing elevated circulating LPS in fibrotic lung diseases.

Interestingly, *Escherichia/Shigella* enrichment has been reported in other autoimmune conditions including inflammatory bowel disease and ankylosing spondylitis (48, 49). Its specific association with RA-ILD in our study, together with the depletion of protective commensals, suggests a model wherein RA-ILD arises from losing beneficial organisms while gaining pro-inflammatory ones. This shifts the gut ecosystem toward a pro-inflammatory state that may facilitate pulmonary involvement through the gut-lung axis.

The absence of significant associations between gut microbial genera and autoantibody status after multiple testing correction warrants discussion. One interpretation is that the gut microbiome’s primary influence in RA-ILD may be on systemic inflammation rather than on autoantibody production per se. Autoantibody generation in RA is strongly linked to genetic factors, particularly HLA-DRB1 shared epitope alleles, and to local citrullination events in the joint and lung (50, 51). While the gut microbiome can influence B-cell responses and antibody production in some contexts (52), its contribution to RA-specific autoantibodies may be modest relative to these other factors. Alternatively, our limited sample size, particularly the small number of seronegative patients, severely constrains statistical power for these comparisons. The trend toward *Escherichia/Shigella* enrichment in seropositive patients observed in uncorrected analyses could emerge as significant in larger, adequately powered cohorts.

Our findings both corroborate and extend the recent work of Xing and colleagues, who performed multi-kingdom metagenomic analysis in a larger cohort (25). Consistent with our results, they reported depletion of *Faecalibacterium prausnitzii* and other SCFA producers in RA-ILD, along with enrichment of certain *Ruminococcus* species. They also identified alterations in fungal and viral communities, aspects not examined in our current analysis. Notably, their larger sample size enabled detection of significant differences in several taxa that we observed only as trends. This highlights the importance of statistical power in microbiome studies. Unlike Xing and colleagues, we did not observe significant enrichment of *Ruminococcus gnavus* in RA-ILD. This discrepancy may reflect differences in population characteristics, disease duration, medication exposure, or technical factors. Such inter-study variability is common in microbiome research and underscores the need for replication across independent cohorts (53).

Several studies have examined gut microbiome alterations in RA without ILD (15, 16, 54). Consistent with our observations in both RA groups, these studies typically report depletion of *Faecalibacterium* and other SCFA producers, along with variable enrichment of *Prevotella copri*. The relative enrichment of *Escherichia/Shigella* that we observed appears more pronounced in our RA-ILD group. This suggests that this may be a feature specifically associated with pulmonary involvement.

Network analysis offers a novel ecological perspective on RA-associated dysbiosis. The hyper-connected microbial network in RA patients shows the disease encourages a community with more microbial interactions, which may amplify inflammatory signals. This pattern has also been found in other chronic inflammatory diseases such as inflammatory bowel disease (55). The partial normalization of network properties in RA-ILD is intriguing; it may indicate that the additional pulmonary involvement imposes a new selective pressure that disrupts some of these interactions, leading to a less complex network. Alternatively, it could reflect a survivor effect, where only a subset of microbial taxa can persist in the face of intensified inflammation or medication changes.

The random forest analysis not only validates the importance of genera like *Escherichia* and *Klebsiella* but also highlights less-studied taxa such as unclassified_Oscillospiraceae and unclassified_Lachnospiraceae as key discriminators. This underscores the need for deeper taxonomic and functional characterization of these under-explored lineages. The high importance of *Bifidobacterium*, a genus typically considered beneficial, is surprising given its reduced abundance in RA; its high feature importance may reflect its role as a strong negative marker of health.

Importantly, the distinct set of top discriminators for RA-ILD versus RA—particularly unclassified_Bacteroidaceae, *Parabacteroides*, and *Blautia*—points to a specific microbial signature associated with ILD development. Members of the *Bacteroidaceae* family, including *Bacteroides* and *Parabacteroides*, are known to modulate host immunity and may contribute to pulmonary inflammation through the gut-lung axis (56, 57). *Blautia* is a butyrate-producing genus whose depletion has been linked to various inflammatory conditions (58); its high importance in our model suggests that loss of this protective organism may be a key event in the progression to ILD. The inclusion of *Ruminococcus* and *Bifidobacterium* further supports the concept that both pro- and anti-inflammatory taxa contribute to the discrimination.

The moderate importance of the Simpson index indicates that while alpha diversity is not significantly different between RA and RA-ILD, the evenness of the community still carries some discriminative information. This aligns with the taxonomic shifts we observed, where certain pro-inflammatory genera expand while others contract, altering the balance of the ecosystem without drastically changing total richness.

The functional genera abundance plots illustrate the complex, sometimes non-linear changes in key microbial players across the disease spectrum. The higher abundance of *Faecalibacterium* in RA-ILD compared to RA, though not statistically significant, is intriguing and warrants further investigation in longitudinal studies. One possible explanation is that the immunosuppressive therapies used to manage ILD may inadvertently promote the regrowth of this butyrate producer, or that the inflammatory milieu in ILD selects for different microbial strains.

Therapeutic speculation: Probiotics (e.g., *Faecalibacterium prausnitzii*, *Roseburia*), prebiotics (e.g., dietary fiber), and postbiotics (e.g., butyrate) possess theoretical potential to restore the balance of gut microbiota, enhance the responses of regulatory T-cells, and mitigate pulmonary inflammation. Nevertheless, these speculations remain unvalidated and necessitate preclinical and clinical verification prior to any recommendations.

Our study has several notable strengths. We employed shotgun metagenomic sequencing, which provides higher taxonomic resolution and functional potential information compared to 16S rRNA amplicon sequencing. Our study design included three well-characterized groups, allowing us to distinguish features specifically associated with ILD from those related to RA itself. We performed comprehensive clinical phenotyping, enabling correlation of microbial features with multiple disease-relevant parameters.

Several limitations must be acknowledged. First, the sample size is modest, limiting statistical power and increasing the risk of false negatives (Type II errors) for smaller effect sizes and false positives (Type I errors) for exploratory uncorrected comparisons. Second, the cross-sectional design precludes causal inference; we cannot determine whether observed microbial alterations precede or follow RA-ILD development. Third, potential confounders including DMARDs (although stable for ≥3 months), immunomodulatory therapy, and unrecorded dietary patterns may influence gut microbiota composition. Fourth, the single-center design and recruitment from a specific geographic region (Suqian, China) may limit generalizability to other populations. Fifth, detailed clinical characterization of RA-ILD (HRCT patterns, pulmonary function tests, disease progression) was not systematically available, precluding stratification by disease severity or subtype. Sixth, our analysis focused on bacterial taxonomy; functional metagenomic profiling (e.g., pathway analysis, metabolite measurement) was not performed, so mechanistic claims about SCFA production or LPS biosynthesis remain speculative and require direct experimental validation.

Despite these limitations, our findings have potential clinical implications. The identification of specific microbial taxa associated with RA-ILD and correlated with disease activity markers raises the possibility of developing microbiome-based biomarkers for early ILD detection in RA patients. A panel combining multiple depleted and enriched taxa might provide diagnostic or prognostic information complementary to existing clinical and radiographic assessments. From a therapeutic perspective, our results suggest that interventions targeting the gut microbiome might have potential in RA-ILD management. Specific strategies could include supplementation with butyrate-producing organisms or their metabolic products, or efforts to reduce *Escherichia/Shigella* colonization. Preclinical evidence supports the feasibility of such approaches. For example, *Faecalibacterium prausnitzii* administration ameliorated colitis in murine models (34), and butyrate supplementation attenuated bleomycin-induced pulmonary fibrosis (40). However, translation of these findings to RA-ILD will require extensive further investigation.

Based on our findings and the limitations discussed, several future research directions emerge. Validation in larger independent cohorts is essential to confirm the taxonomic associations we observed and to identify additional RA-ILD-specific microbial features. Longitudinal studies following RA patients from early disease through ILD development could establish temporal relationships and identify predictive microbial signatures. Beyond taxonomy, characterizing microbial gene content and actual metabolic products would provide deeper mechanistic insights. Comprehensive analysis including bacterial, fungal, and viral components may yield more robust signatures. Examining interactions among gut microbiota, host genetic risk factors, and immune phenotypes could illuminate mechanisms linking gut dysbiosis to pulmonary fibrosis. Preclinical models testing microbiome-targeted interventions in RA-ILD-relevant systems, followed by carefully designed clinical trials, could establish causality and explore therapeutic potential.

This metagenomic study demonstrates that RA-ILD is associated with a distinct gut microbial “dichotomous” imbalance—enrichment of the pro-inflammatory genus *Escherichia/Shigella* and depletion of butyrate-producing genera *Roseburia* and *Ruminococcus*—despite preserved overall microbial diversity. These taxonomic shifts correlated robustly with systemic inflammation and disease activity, providing new clinical evidence for the gut–lung axis in RA-ILD. The study further reveals that RA is characterized by a hyper-connected microbial ecological network that partially reverts to a healthier topology in the presence of ILD, suggesting dynamic microbial adaptation under disease pressure. Using machine learning, we identified specific bacterial panels (*Bifidobacterium*, unclassified_Oscillospiraceae, unclassified_Lachnospiraceae for HC vs. RA; unclassified_Bacteroidaceae, *Parabacteroides*, and *Blautia* for RA-ILD vs. RA) that may serve as candidate biomarkers for disease diagnosis and risk stratification. Collectively, these findings highlight the potential of targeting gut microbiota to monitor and manage RA-ILD, and underscore the need for larger, longitudinal studies to establish causality and translate these microbial signatures into clinical practice.

## Data Availability

Raw metagenomic sequencing data have been deposited in the NCBI Sequence Read Archive under accession number PRJNA1441104. Additional data supporting the findings of this study are available from the corresponding author upon reasonable request.
