# Supplementary material for: Metagenomic characterization of gut microbiota in rheumatoid arthritis-associated interstitial lung disease: taxonomic shifts and clinical correlations
Source: Front Immunol. 2026 Jun 12;17:1868704. doi: 10.3389/fimmu.2026.1868704 (PMC13303103; doi:10.3389/fimmu.2026.1868704)
Supplement: Supplementary file 1 [file Image1.pdf]

Supplementary Figure S1. Phylum-Level Composition Across Groups.

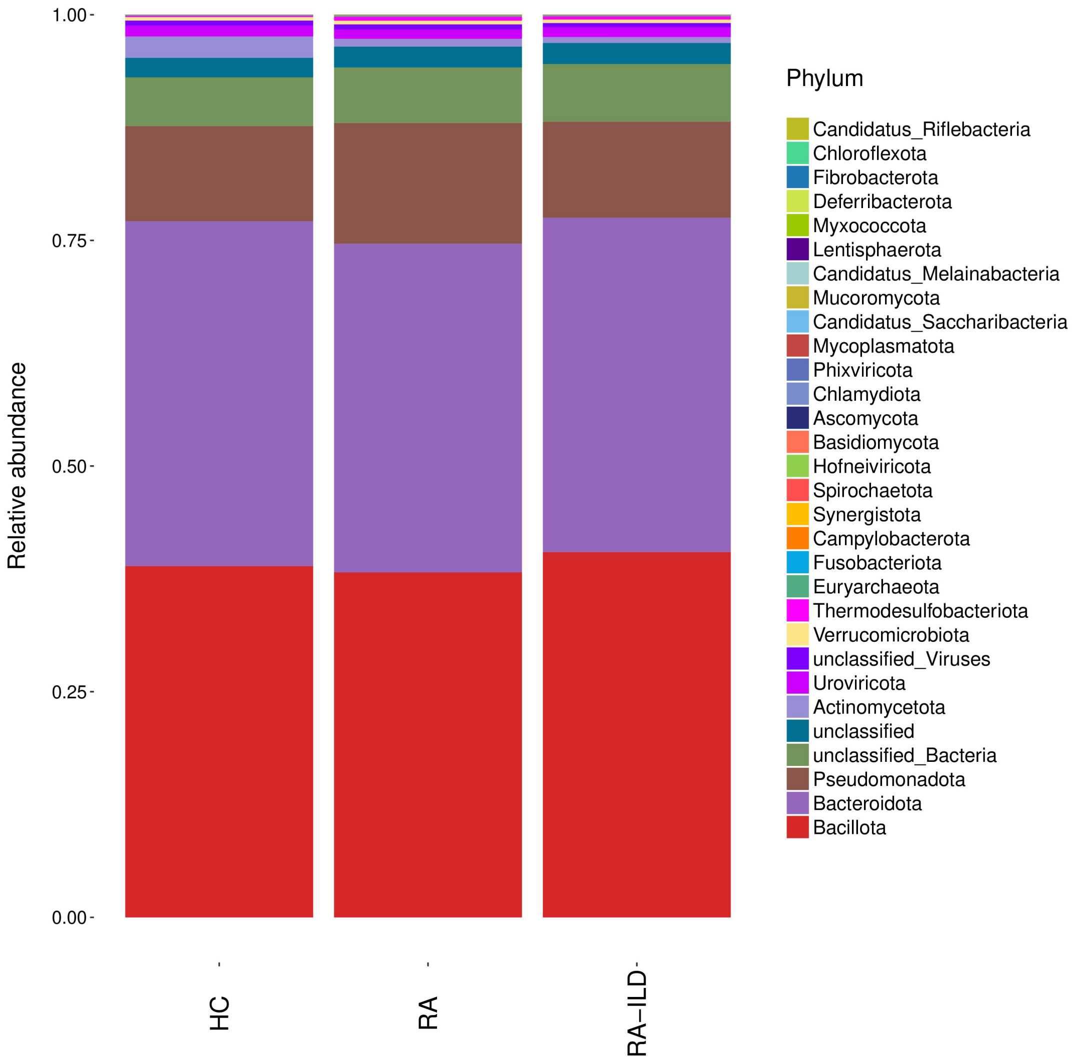

Figure S1. Phylum-level relative abundance of gut microbiota in HC (n=11), RA (n=20), and RA-ILD (n=10) groups. Only phyla with mean relative abundance ≥1% are shown; remaining phyla are grouped as "Others". The composition is dominated by *Bacillota* (formerly Firmicutes), *Bacteroidota*, *Pseudomonadota*, and *Actinomycetota*, consistent with typical human gut microbiome profiles.

Abbreviations: HC, healthy control; RA, rheumatoid arthritis; RA-ILD, rheumatoid arthritis-associated interstitial lung disease.
