# Supplementary material for: Metagenomic characterization of gut microbiota in rheumatoid arthritis-associated interstitial lung disease: taxonomic shifts and clinical correlations
Source: Front Immunol. 2026 Jun 12;17:1868704. doi: 10.3389/fimmu.2026.1868704 (PMC13303103; doi:10.3389/fimmu.2026.1868704)
Supplement: Supplementary file 2 [file Image2.pdf]

Supplementary Figure S2. Genus-Level Composition Across Groups

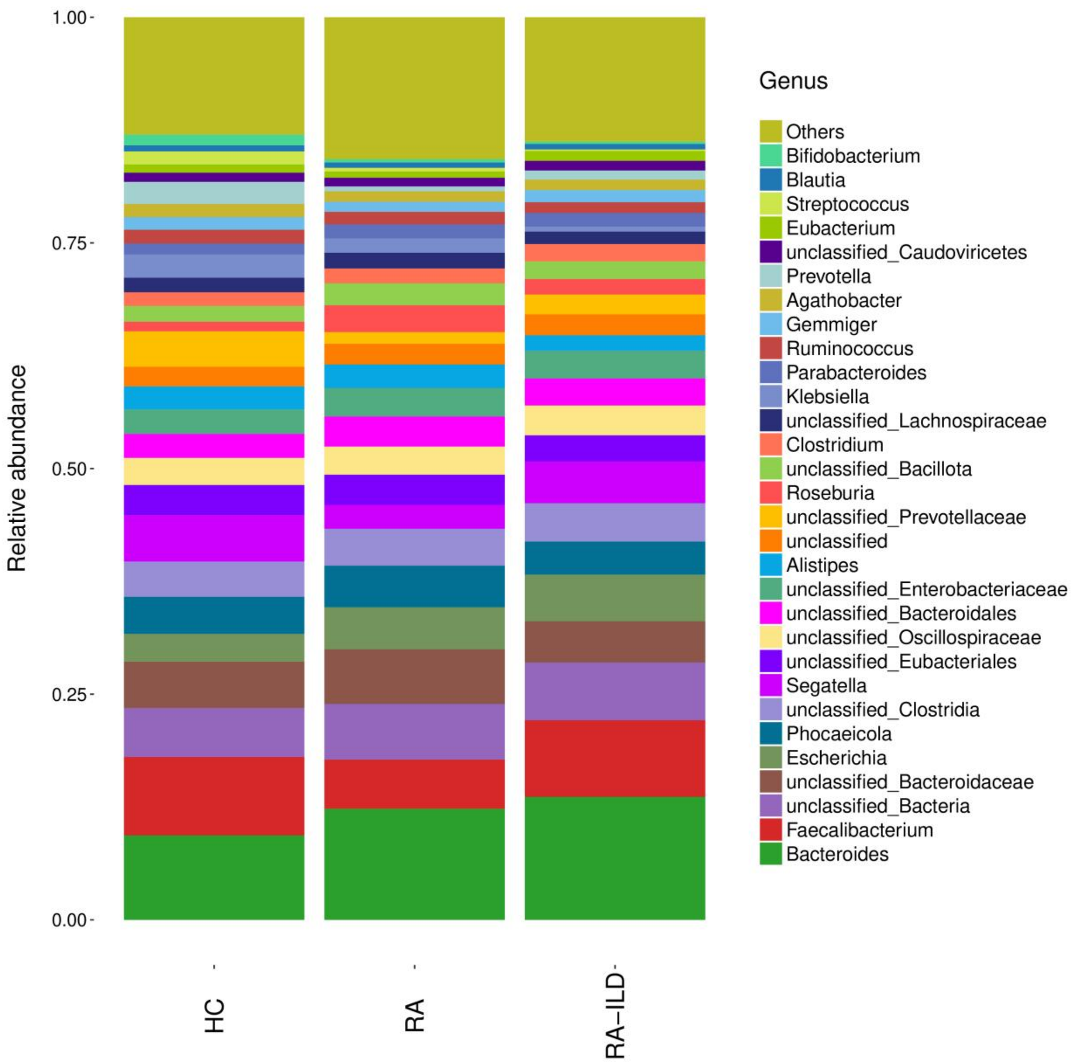

Figure S2. Genus-level relative abundance of gut microbiota in HC (n=11), RA (n=20), and RA-ILD (n=10) groups. Only the top 15 most abundant genera are shown; remaining genera are grouped as "Others". *Bacteroides*, *Faecalibacterium*, *Prevotella*, and *Escherichia* were the most abundant taxa, with substantial inter-individual variation.

Abbreviations: HC, healthy control; RA, rheumatoid arthritis; RA-ILD, rheumatoid arthritis-associated interstitial lung disease.
