# Supplementary material for: Metagenomic characterization of gut microbiota in rheumatoid arthritis-associated interstitial lung disease: taxonomic shifts and clinical correlations
Source: Front Immunol. 2026 Jun 12;17:1868704. doi: 10.3389/fimmu.2026.1868704 (PMC13303103; doi:10.3389/fimmu.2026.1868704)
Supplement: Supplementary file 3 [file Image3.pdf]

Supplementary Figure S3. Confusion matrix of the random forest classifier for HC vs. RA.

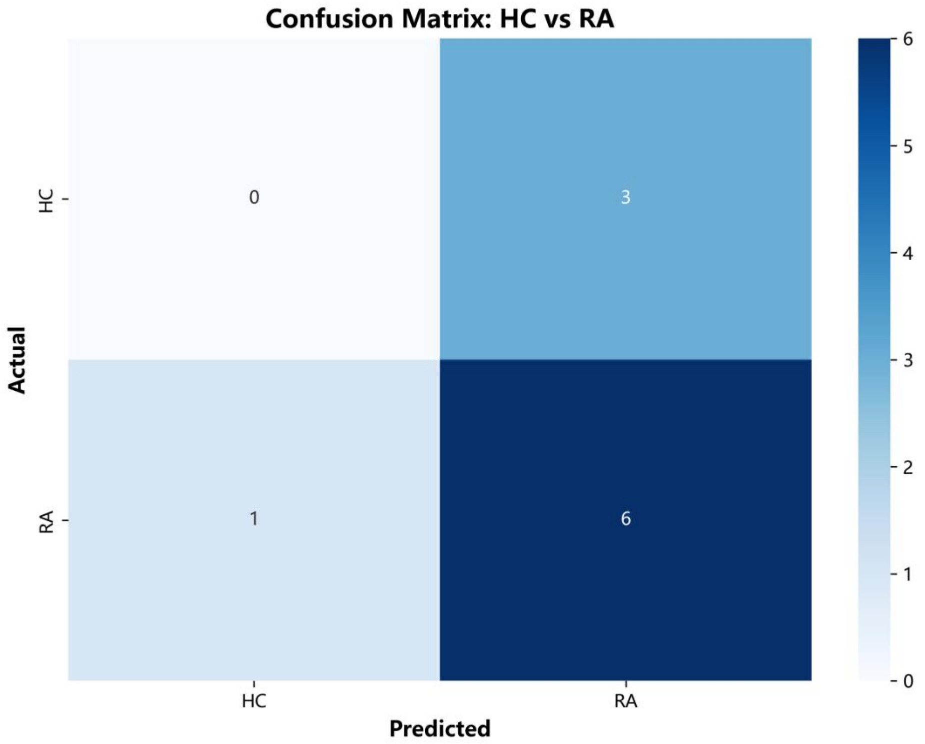

Figure S3. Confusion matrix of the random forest classifier trained to distinguish healthy controls (HC) from rheumatoid arthritis (RA) patients based on genus-level relative abundances and alpha diversity indices. The model achieved a cross-validated AUC of 0.96. Values in the matrix represent the number of samples correctly (diagonal) or incorrectly (off-diagonal) classified.

Abbreviations: HC, healthy control; RA, rheumatoid arthritis.
