# Supplementary material for: Metagenomic characterization of gut microbiota in rheumatoid arthritis-associated interstitial lung disease: taxonomic shifts and clinical correlations
Source: Front Immunol. 2026 Jun 12;17:1868704. doi: 10.3389/fimmu.2026.1868704 (PMC13303103; doi:10.3389/fimmu.2026.1868704)
Supplement: Supplementary file 4 [file Image4.pdf]

**Supplementary Figure S4. Confusion matrix of the random forest classifier for RA- ILD vs. RA.**

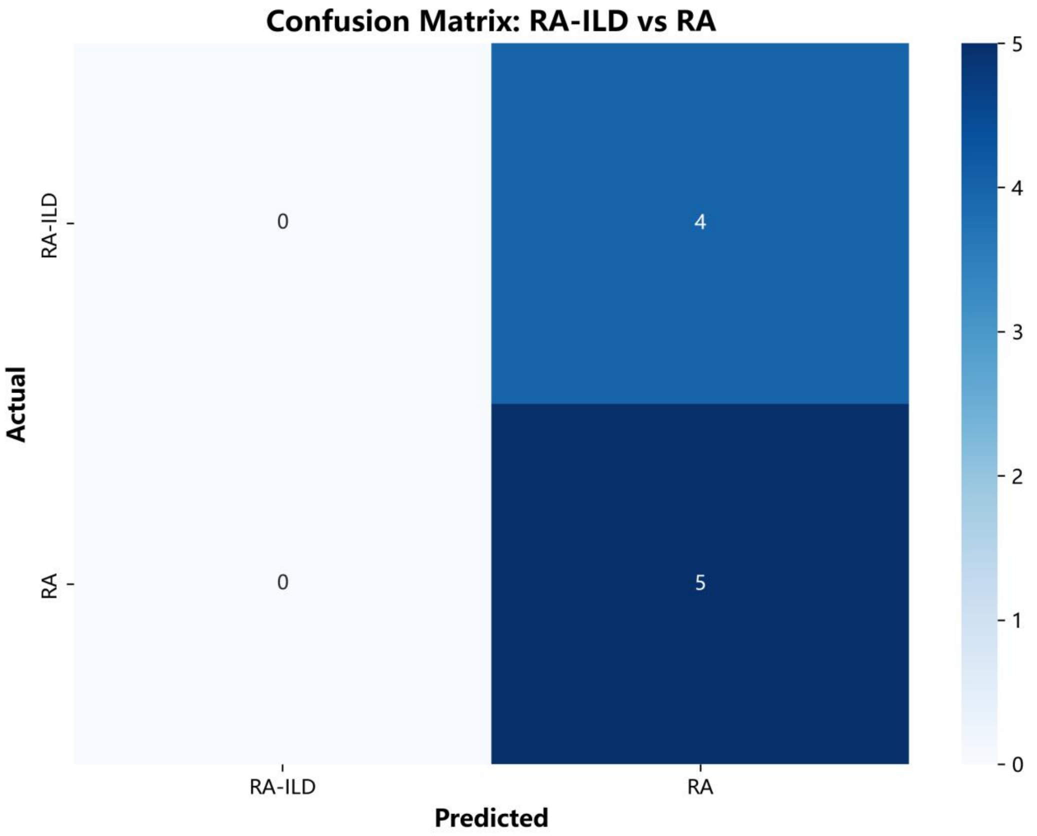


**Figure S4.** Confusion matrix of the random forest classifier trained to distinguish rheumatoid arthritis- associated interstitial lung disease (RA- ILD) from rheumatoid arthritis (RA) patients based on genus- level relative abundances and alpha diversity indices. The model achieved a cross- validated accuracy of 82%. Values in the matrix represent the number of samples correctly (diagonal) or incorrectly (off- diagonal) classified.

**Abbreviations:** RA, rheumatoid arthritis; RA- ILD, rheumatoid arthritis- associated interstitial lung disease.
