# Supplementary material for: Metagenomic characterization of gut microbiota in rheumatoid arthritis-associated interstitial lung disease: taxonomic shifts and clinical correlations
Source: Front Immunol. 2026 Jun 12;17:1868704. doi: 10.3389/fimmu.2026.1868704 (PMC13303103; doi:10.3389/fimmu.2026.1868704)
Supplement: Supplementary file 5 [file Table1.pdf]

Supplementary Table S1. **Sequencing Quality Metrics by Group**

Sample	Length(nt)	#Reads	#Bases	Q20(%)	Q30(%)	GC(%)	N(ppm)
HC-1	148.77	71559008	10646076643	98.25	94.92	49.36	2.33
HC-2	148.63	77834376	11568637858	98.21	94.81	47.64	2.31
HC-3	149.27	72402332	10807646657	98	94.35	46.36	2.29
HC-4	148.47	75190844	11163583644	98.31	95.13	51.22	2.29
HC-5	148.82	75023068	11165109530	98.29	94.95	45.93	2.33
HC-6	148.06	74153024	10979065602	98.39	95.42	53.94	2.45
HC-7	148.77	77251528	11492923849	98.16	94.63	47.92	2.29
HC-8	148.65	78689578	11697399602	98.03	94.46	50.84	2.29
HC-9	148.71	76545742	11383099137	98.14	94.75	51.46	2.37
HC-10	148.83	73204866	10894920598	98.27	94.93	46.55	2.38
HC-11	148.62	72121614	10718808149	98.17	94.73	49.18	2.21
RA-1	147.99	75809470	11219249514	98.38	95.12	47.79	2.34
RA-2	148.8	76273888	11349203452	98.25	94.81	46.82	2.31
RA-3	148.83	72171504	10740928585	98.21	94.69	45.9	2.33
RA-4	149.09	73411170	10944958521	98.23	94.89	50.14	2.25
RA-5	148.68	75437650	11216069528	98.11	94.69	52.83	2.36
RA-6	148.55	74855232	11119645162	98.1	94.54	49.15	2.37
RA-7	148.44	77989730	11577173245	98.21	94.74	48	2.3
RA-8	149.12	75034988	11189050309	98.32	94.95	47.13	2.31
RA-9	149.09	72678980	10835649254	98.12	94.51	48.15	2.32
RA-10	148.63	78414934	11654826797	98.24	94.8	47.19	2.37
RA-11	148.62	74785176	11114234526	98.33	95.01	47	2.34
RA-12	149.35	74827824	11175663586	97.83	93.74	44.47	2.32
RA-13	149.07	76982466	11475863396	98.31	94.96	47.28	2.31
RA-14	148.92	74484056	11092468848	98.2	94.66	42.98	2.3
RA-15	149.05	77871158	11606890881	98.14	94.65	50.39	2.38
RA-16	148.96	76654010	11418054466	98.11	94.56	48.15	2.36
RA-17	148.8	73035700	10867419215	98.21	94.93	52.38	2.24
RA-18	148.79	74487490	11083201939	98.02	94.37	48.99	2.32
RA-19	149.06	73825784	11004406299	98.29	94.91	46.91	2.25
RA-20	148.95	77158358	11492961915	98.24	94.88	50.32	2.28
RA-ILD-1	148.93	76086906	11331694950	98.25	94.94	49.08	2.33
RA-ILD-2	148.66	78008908	11596693817	98	94.37	50.16	2.33
RA-ILD-3	148.64	76468600	11366003987	98.33	95.03	48.11	2.33
RA-ILD-4	148.74	72409618	10770279494	98.3	94.93	47.3	2.26
RA-ILD-5	148.91	73484948	10943001131	98.25	94.89	48.13	2.31
RA-ILD-6	148.78	73802384	10980530238	98.16	94.72	49.74	2.29
RA-ILD-7	148.63	76671042	11395325837	98.19	94.8	50.2	2.26
RA-ILD-8	148.99	72376800	10783300734	98.28	94.99	48.76	2.31
RA-ILD-9	148.82	77047872	11466232667	98.32	94.93	45.38	2.32
RA-ILD-10	149.19	75918598	11326669946	98.25	94.71	44.33	2.31

Note: Sequencing quality metrics after quality control (QC). Length(nt) is the average read length; #Reads is the total number of reads; #Bases is the total number of bases; Q20(%) and Q30(%) are the percentages of bases with Phred quality scores >20 and >30, respectively; GC(%) is the percentage of G and C bases; N(ppm) is the number of ambiguous bases per million bases. All samples showed high quality with Q30 > 93.7%.
