# Supplementary material for: Metagenomic characterization of gut microbiota in rheumatoid arthritis-associated interstitial lung disease: taxonomic shifts and clinical correlations
Source: Front Immunol. 2026 Jun 12;17:1868704. doi: 10.3389/fimmu.2026.1868704 (PMC13303103; doi:10.3389/fimmu.2026.1868704)
Supplement: Supplementary file 6 [file Table2.pdf]

Supplementary Table S2. Genus-Level Abundances in HC and RA-ILD Groups with Statistical Comparisons.

Genus	HC_mean	HC_sd	RAILD_mean	RAILD_sd	P_value	FDR_q
Bacteroides	625971.68	641461.69	892064.15	920039.60	0.751334313	0.929220197
Faecalibacterium	585319.25	416697.55	530171.34	426454.71	0.751334313	0.929220197
unclassified_Bacteria	362511.95	156937.89	411370.87	156763.65	0.459671591	0.929220197
unclassified_Bacteroidaceae	345198.06	250249.93	291419.47	305832.23	0.503514710	0.929220197
Phocaeicola	273640.35	226181.15	233766.85	284758.40	0.459671591	0.929220197
unclassified_Clostridia	262538.24	111735.36	275626.05	248553.38	0.503514710	0.929220197
Segatella	337416.97	490257.71	270277.54	487503.19	0.647156338	0.929220197
unclassified_Eubacteriales	224861.15	154489.38	185680.88	121257.69	0.647156338	0.929220197
unclassified_Oscillospiraceae	202562.06	114304.57	209073.45	125996.60	0.805324171	0.929220197
unclassified_Bacteroidales	177699.06	87198.32	192433.83	128396.62	0.971913134	1.000000000
unclassified_Enterobacteriaceae	185014.03	216029.01	200198.97	308541.11	0.751334313	0.929220197
Alistipes	173519.88	155180.76	107028.25	99090.09	0.549471692	0.929220197
unclassified	145889.29	38150.59	148198.33	44129.96	0.549471692	0.929220197
Roseburia	70727.36	43345.62	**111284.85**	86397.18	0.307226370	0.929220197
unclassified_Bacillota	118718.49	68119.35	124534.72	80956.54	0.805324171	0.929220197
unclassified_Prevotellaceae	252966.88	351009.25	128517.36	226204.00	0.307226370	0.929220197
Clostridium	101697.37	52059.62	125003.08	154402.40	0.597406270	0.929220197
unclassified_Lachnospiraceae	108309.58	37198.49	88912.11	53224.29	0.192666696	0.929220197
Klebsiella	175472.33	332508.98	33859.06	38726.21	0.805324171	0.929220197
Parabacteroides	81449.08	46944.60	99035.99	104004.14	0.549471692	0.929220197
Ruminococcus	100837.63	83064.94	75073.68	78650.59	0.307226370	0.929220197
Gemmiger	93589.92	66184.90	85765.26	70605.55	0.805324171	0.929220197
Agathobacter	96830.35	106874.25	73362.08	88204.61	0.597406270	0.929220197

Genus	HC_mean	HC_sd	RAILD_mean	RAILD_sd	P_value	FDR_q
Prevotella	154641.33	310399.25	60425.15	92660.76	0.647156338	0.929220197
unclassified_Caudoviricetes	68009.23	26857.04	66816.29	35814.90	0.698535358	0.929220197
Eubacterium	62327.86	64237.16	70117.87	60063.39	0.597406270	0.929220197
Blautia	44041.19	33176.85	38964.73	28811.52	0.915878446	0.981298335
unclassified_Viruses	38192.27	25850.62	29310.31	20996.99	0.192666696	0.929220197
Dialister	18275.32	27811.40	45409.92	91545.95	0.915878446	0.981298335
Escherichia/Shigella	216465.72	376644.24	348616.99	555341.78	1.000000000	1.000000000

Note: Data are presented as mean ± standard deviation (SD) of absolute abundances (MetaPhlAn4- derived abundance scores, not percentages). Statistical comparisons between HC and RA- ILD groups were performed using Wilcoxon rank- sum tests. P_value is the raw *P* value; FDR_q is the Benjamini- Hochberg false discovery rate adjusted q- value. *Escherichia/Shigella* showed a trend toward higher abundance in RA- ILD, but the difference was not statistically significant (*P* = 1.000, q = 1.000).
