# Supplementary material for: Metagenomic characterization of gut microbiota in rheumatoid arthritis-associated interstitial lung disease: taxonomic shifts and clinical correlations
Source: Front Immunol. 2026 Jun 12;17:1868704. doi: 10.3389/fimmu.2026.1868704 (PMC13303103; doi:10.3389/fimmu.2026.1868704)
Supplement: Supplementary file 7 [file Table3.pdf]

**Supplementary Table S3. Spearman correlation coefficients between core gut microbial genera and clinical parameters in RA patients (n = 30).**

| | Disease_duration,years | Age,years | ESR,mm/h | CRP,mg/L | DAS28-ESR | DAS28-CRP | SDAI | CDAI | RF_positive | Anti-CCP_positive |
|---|---|---|---|---|---|---|---|---|---|---|
| Bacteroides | 0.115480049 | -0.050373433 | -0.015576324 | 0.219229935 | -0.105919006 | 0.147942158 | 0.115016685 | -0.068812059 | 0.211840812 | 0.022658132 |
| Faecalibacterium | -0.136923153 | -0.033879477 | -0.177792617 | -0.305141361 | -0.143079665 | -0.33570634 | -0.372191324 | -0.220287954 | -0.067169038 | 0.16993599 |
| unclassified_Bacteria | 0.132232474 | 0.157807037 | 0.352915007 | 0.439572712 | 0.236982649 | 0.306785317 | 0.319243604 | 0.131815307 | 0.418514774 | 0.237910386 |
| unclassified_Bacteroidaceae | -0.231630195 | -0.095174583 | 0.081664443 | 0.112397073 | -0.060747665 | 0.160400445 | 0.143937709 | 0.039321176 | -0.211840812 | -0.237910386 |
| Escherichia | 0.322986752 | 0.176975688 | 0.234312422 | 0.281549104 | 0.390520704 | 0.225806452 | 0.285873192 | 0.240395374 | 0.304844095 | 0.28322665 |
| Phocaeicola | -0.314498856 | -0.188566036 | 0.095905654 | 0.113732484 | -0.050289276 | 0.102113459 | 0.083426029 | -0.021447914 | -0.232508208 | -0.181265056 |
| unclassified_Clostridia | -0.265135044 | -0.107879387 | -0.285714293 | -0.225461852 | -0.499109937 | -0.258286986 | -0.305450501 | -0.199733703 | -0.242841906 | -0.203923188 |
| unclassified_Bacteroidales | 0.05874517 | -0.077343279 | -0.11326213 | 0.180948159 | -0.132398757 | 0.097664071 | 0.062958843 | -0.124442587 | 0.211840812 | 0.05664533 |
| unclassified_Eubacteriales | 0.025910417 | 0.325644182 | -0.107921676 | -0.232138906 | -0.16688919 | -0.255617353 | -0.144382647 | -0.106792741 | -0.046501642 | 0.124619726 |
| unclassified_Oscillospiraceae | -0.108555713 | 0.228017795 | -0.131953719 | -0.331849577 | -0.164886519 | -0.296551724 | -0.232035595 | -0.121538182 | 0.036167943 | 0.05664533 |
| unclassified_Enterobacteriaceae | 0.234310583 | 0.130391408 | 0.33978639 | 0.33719122 | 0.377169569 | 0.2467185 | 0.29032 | 0.2222986 | 0.242841906 | 0.158606924 |

| | | | | | | | | | | |
|---|---|---|---|---|---|---|---|---|---|---|
| | | | | | | 76 | 2581 | 96 | | |
| Segatella | 0.001116828 | -0.01381926 | -0.024032043 | 0.005119075 | -0.031375168 | -0.057174638 | -0.088320356 | -0.006702473 | -0.180839717 | -0.067974396 |
| Roseburia | -0.417917159 | 0.258999685 | -0.151535384 | -0.240596507 | -0.427458845 | -0.518576196 | -0.437152392 | -0.439235414 | 0.10850383 | 0.05664533 |
| unclassified | 0.052490931 | 0.17207208 | 0.206275038 | 0.191186309 | -0.186025817 | -0.093659622 | -0.054949944 | -0.308537185 | 0.346178887 | 0.11329066 |
| Alistipes | -0.148761533 | 0.145102233 | -0.039830887 | 0.078566667 | -0.113707168 | -0.090989989 | -0.032703003 | -0.090259973 | -0.067169038 | 0.05664533 |
| unclassified_Bacillota | 0.052490931 | 0.267023771 | -0.108144195 | -0.169819736 | -0.170672011 | -0.241824249 | -0.130144605 | -0.096068783 | 0.129171227 | 0.11329066 |
| Clostridium | -0.048693715 | -0.171626297 | -0.271028044 | -0.119519264 | -0.247886076 | -0.009566185 | -0.099443826 | 0.008936631 | -0.346178887 | -0.203923188 |
| unclassified_Lachnospiraceae | -0.403845122 | 0.002451804 | -0.19359146 | -0.237925686 | -0.392968412 | -0.402447164 | -0.355728587 | -0.321942132 | -0.149838623 | -0.192594122 |
| Parabacteroides | 0.092473385 | 0.093614344 | -0.008010681 | 0.331849577 | -0.034267914 | 0.238709677 | 0.227141268 | 0.031054793 | 0.346178887 | 0.11329066 |
| unclassified_Prevotellaceae | -0.003350485 | -0.02072889 | -0.137516692 | -0.074560435 | -0.05140187 | -0.097664071 | -0.136373749 | 0.01005371 | -0.211840812 | 0 |
| Ruminococcus | -0.378381437 | 0.166722689 | 0.002670227 | -0.259737395 | -0.238317763 | -0.329477197 | -0.250278087 | -0.174040889 | -0.253175604 | -0.373859179 |
| Klebsiella | 0.2081768 | 0.138415494 | 0.319759687 | 0.187180076 | 0.238762801 | 0.186651835 | 0.200889878 | 0.107686404 | 0.232508208 | -0.045316264 |
| Gemmiger | -0.060978827 | 0.36487305 | 0.011570984 | -0.16492323 | -0.398531385 | -0.442046719 | -0.32057842 | -0.420468489 | 0.180839717 | -0.011329066 |
| Agathobacter | -0.267368701 | 0.044132476 | -0.278816206 | -0.295348349 | -0.25122386 | -0.385984 | -0.3828 | -0.259832 | 0.05683534 | 0.045316264 |

| | | | | | | | | | | |
|---|---|---|---|---|---|---|---|---|---|---|
| | | | | | | 427 | 69855 | 546 | | |
| unclassified_Caudoviricetes | 0.057851707 | 0.133511886 | -0.118380065 | -0.152904533 | -0.097240768 | -0.183982202 | -0.115016685 | -0.165551089 | 0.036167943 | 0.079303462 |
| Eubacterium | -0.500785821 | 0.191909405 | -0.204272368 | -0.379924364 | -0.350912336 | -0.524805339 | -0.501223582 | -0.345177373 | 0.025834245 | 0.147277858 |
| Prevotella | -0.028144074 | 0.063524019 | -0.079439254 | 0.030937016 | -0.005117935 | -0.066963293 | -0.076307008 | -0.015415688 | -0.201507114 | -0.033987198 |
| Blautia | -0.127095063 | -0.291987596 | -0.171784606 | -0.24460274 | -0.066310638 | -0.094549499 | -0.167964405 | -0.014968857 | -0.356512585 | -0.249239452 |
| Dialister | -0.079741542 | 0.094505909 | -0.060080108 | -0.284665063 | -0.326435255 | -0.363292547 | -0.391323693 | -0.263630615 | -0.067169038 | -0.249239452 |
| unclassified_Viruses | 0.098727624 | 0.177867253 | -0.160213622 | -0.104384609 | 0.176234984 | 0.004226919 | 0.067408231 | 0.129804565 | 0.025834245 | 0.22658132 |

**Note:** Values are Spearman's rank correlation coefficients (ρ) between the relative abundance of each genus and clinical parameters in RA patients (n = 30). Disease_duration is in years; ESR, erythrocyte sedimentation rate (mm/h); CRP, C-reactive protein (mg/L); DAS28-ESR and DAS28-CRP, Disease Activity Score in 28 joints; SDAI, Simplified Disease Activity Index; CDAI, Clinical Disease Activity Index; RF_positive and Anti-CCP_positive are binary variables (1 = positive, 0 = negative). The heatmap of this correlation matrix is presented in Figure 2A. Corresponding *P*-values are provided in Supplementary Table S4.
