# Supplementary material for: Metagenomic characterization of gut microbiota in rheumatoid arthritis-associated interstitial lung disease: taxonomic shifts and clinical correlations
Source: Front Immunol. 2026 Jun 12;17:1868704. doi: 10.3389/fimmu.2026.1868704 (PMC13303103; doi:10.3389/fimmu.2026.1868704)
Supplement: Supplementary file 8 [file Table4.pdf]

Supplementary Table S4. Corresponding P-values for the Spearman correlation coefficients shown in Supplementary Table S3.

	**Disease_duration,years**	**Age,years**	**ESR,mm/h**	**CRP,mg/L**	**DAS-ESR**	**DAS-CRP**	**SDAI**	**CDAI**	**RF_positive**	**Anti-CCP_positive**
Bacteroides	0.543404698	0.791509733	0.934889533	0.244433644	0.577484997	0.433644892	0.543538332	0.717865565	0.261093541	0.90539787
Faecalibacterium	0.470601327	0.858933988	0.347243371	0.10107063	0.450685046	0.070265443	0.043598814	0.242107307	0.724338849	0.369301645
unclassified_Bacteria	0.486078162	0.404926765	0.055757611	0.015077386	0.207342385	0.099376119	0.085875396	0.487467075	0.021352389	0.205516704
unclassified_Bacteroidaceae	0.218092542	0.616877727	0.667918746	0.554289868	0.749813603	0.395538871	0.446311897	0.836557331	0.261093541	0.205516704
Escherichia	0.08170018	0.349499296	0.212659053	0.131743551	0.032873184	0.229241945	0.125605838	0.200681022	0.101420217	0.129360477
Phocaeicola	0.090523511	0.318317183	0.614162347	0.549562527	0.791850464	0.589949222	0.660090185	0.910430079	0.216303639	0.337752408
unclassified_Clostridia	0.156779442	0.570420315	0.12588507	0.230943508	0.004989102	0.16769658	0.100913722	0.289959444	0.195997409	0.279749896
unclassified_Bacteroidales	0.757812359	0.684569571	0.55122543	0.338611964	0.485525102	0.606349169	0.740325246	0.512344046	0.261093541	0.766226343
unclassified_Eubacteriales	0.891892842	0.079077945	0.570268346	0.217054851	0.378072435	0.172251898	0.444894548	0.574331485	0.807223696	0.511739101
unclassified_Oscillospiraceae	0.567992086	0.225558209	0.487006022	0.073206812	0.383902951	0.111632545	0.216386031	0.522312826	0.849509566	0.766226343
unclassified_Enterobacteriaceae	0.212662748	0.492223017	0.066195422	0.06842792	0.039913233	0.188076066	0.119632988	0.237726849	0.195997409	0.402519298
Segatella	0.995326671	0.9422213	0.899689366	0.978581729	0.869269069	0.763545951	0.641408241	0.971959044	0.338906409	0.721163485
Roseburia	0.021557798	0.166960368	0.424082924	0.200293089	0.018465718	0.003755939	0.016494798	0.015164178	0.568178196	0.766226343
unclassified	0.78294904	0.363224472	0.274121083	0.311514507	0.324999104	0.62127409	0.772532926	0.097141232	0.060937975	0.551124497
Alistipes	0.432712399	0.444242009	0.834467478	0.679840329	0.54965197	0.631308328	0.863833212	0.635259106	0.724338849	0.766226343
unclassified_Bacillota	0.78294904	0.153736854	0.569469012	0.369634092	0.367200953	0.197205023	0.491452978	0.613557126	0.496317154	0.551124497
Clostridium	0.798317549	0.364487813	0.14742706	0.529297403	0.186581145	0.960659573	0.599765692	0.962617717	0.060937975	0.279749896
unclassified_Lachnospiraceae	0.026884359	0.989740723	0.305350727	0.205486689	0.031698814	0.028300806	0.054385431	0.082749023	0.429350213	0.3078973
Parabacteroides	0.626953444	0.622689397	0.966488724	0.073206812	0.857332941	0.203173799	0.226444188	0.870592792	0.060937975	0.551124497
unclassified_Prevotellaceae	0.98598062	0.913421476	0.468661463	0.695370591	0.787348896	0.606349169	0.47078081	0.957948797	0.261093541	1
Ruminococcus	0.039228168	0.378555206	0.988826814	0.165711998	0.204718532	0.075886046	0.181627423	0.357675949	0.177051122	0.041833571
Klebsiella	0.269623363	0.465731894	0.084973541	0.321952262	0.203849009	0.32189973	0.285821273	0.571114043	0.216303639	0.812049687

Gemmiger	0.748891871	0.047420809	0.951609441	0.383795606	0.029155937	0.015224037	0.084517578	0.020692077	0.338906409	0.952620019
Agathobacter	0.153185814	0.816875949	0.13569444	0.113072615	0.180526773	0.035955449	0.03757797	0.165551465	0.765463891	0.812049687
unclassified_Caudoviricetes	0.761389179	0.481831138	0.533258248	0.419858824	0.609216308	0.328968526	0.543538332	0.381962401	0.849509566	0.676997826
Eubacterium	0.004822344	0.309653304	0.278909525	0.038369641	0.057260132	0.003307217	0.005293379	0.061739331	0.89220882	0.437367254
Prevotella	0.882634553	0.738765853	0.676474422	0.871079506	0.978586495	0.724380097	0.68762848	0.935559616	0.285609179	0.85848993
Blautia	0.503322657	0.117426717	0.364038871	0.192673703	0.727728287	0.617944201	0.373384049	0.937423775	0.0531366	0.184109413
Dialister	0.675309799	0.619365692	0.752477309	0.127342435	0.078309982	0.049191382	0.03330712	0.159233648	0.724338849	0.184109413
unclassified_Viruses	0.603727937	0.347037702	0.397708114	0.58304156	0.35155231	0.983134457	0.722615341	0.494189939	0.89220882	0.228574291

Note: Data represent raw *P*-values prior to multiple testing correction, rounded to three decimal places. Values <0.001 are indicated as "<0.001". Bold *P*-values indicate nominal statistical significance ($P < 0.05$). No associations remained significant after Benjamini-Hochberg FDR correction (see Table 3 and main text).
