# Supplementary material for: Metagenomic characterization of gut microbiota in rheumatoid arthritis-associated interstitial lung disease: taxonomic shifts and clinical correlations
Source: Front Immunol. 2026 Jun 12;17:1868704. doi: 10.3389/fimmu.2026.1868704 (PMC13303103; doi:10.3389/fimmu.2026.1868704)
Supplement: Supplementary file 9 [file Table5.pdf]

Supplementary Table S5. Associations with RF and anti-CCP Seropositivity

Genus	RF_neg_mean	RF_neg_sd	RF_pos_mean	RF_pos_sd	P_value.x	ACPA_neg_mean	ACPA_neg_sd	ACPA_pos_mean	ACPA_pos_sd	P_value.y	RF_FDR	ACPA_FDR
Bacteroides	379808.88	178967.66	926825.196	751904.57	0.275	618045.975	431019.778	869134.246	752798.952	0.930	0.55	0.996
Faecalibacterium	450613.68	422432.313	408889.496	365554.72	0.746	235743.35	261773.131	443551.246	378042.095	0.391	0.914	0.96
unclassified_Bacteria	269550.26	92034.283	433590.392	149444.537	0.022	320125.325	75291.243	419500.377	158842.034	0.022	0.391	0.96
unclassified_Bacteroidaceae	485950.78	333451.724	334233.08	275909.235	0.275	526094.6	333619.361	333892.404	276095.725	0.220	0.55	0.96
Escherichia	72448.9	134500.063	366484.356	484871.279	0.108	28734.15	33900.544	361900.646	476938.331	0.139	0.541	0.96
Phocaeicola	473340.22	382783.713	237964.488	200017.163	0.229	458801.7	325668.304	249254.096	228621.719	0.359	0.55	0.96
unclassified_Clostridia	314873.2	105288.48	259431.624	174162.035	0.208	315218.125	118024.227	261510.927	171270.47	0.298	0.55	0.96
Segatella	271179.04	304662.151	186453.016	365920.882	0.355	176347.75	336114.74	204301.138	361834.91	0.746	0.592	0.966
unclassified_Eubacteriales	200257.2	151123.56	209814.396	180891.714	0.829	259052.875	363004.365	200401.323	137086.184	0.536	0.914	0.96
unclassified_Oscillospiraceae	178598.38	110069.851	210607.056	188186.751	0.872	304508.075	386879.685	190005.231	127780.084	0.791	0.914	0.966
unclassified_Bacteroidales	145243.4	37382.551	221705.648	129829.328	0.275	192967.575	127452.22	211422.612	124027.888	0.791	0.55	0.966
unclassified_Enterobacteriaceae	113792.1	164260.974	222871.156	274342.22	0.208	108325.125	175735.185	219516.881	270430.863	0.425	0.55	0.96
Alistipes	162175.74	138433.909	146188.888	148373.166	0.746	108981.6	87070.426	154987.481	151787.996	0.791	0.914	0.966
unclassified	120687.4	20973.272	155490.592	41772.505	0.065	142696.575	55168.268	150765.981	39662.932	0.576	0.391	0.96
Roseburia	80190.92	75314.296	183725.176	278159.866	0.589	96739.825	102715.524	177197.104	273511.265	0.791	0.841	0.966
unclassified_Bacillota	108012.54	55940.446	156160.24	116713.56	0.516	149260.325	160792.385	147962.592	104218.505	0.576	0.774	0.96
unclassified_Prevotellaceae	149509.76	144379.452	86565.984	164744.665	0.275	74116.55	138712.604	100585.854	166266.848	1	0.55	1
Clostridium	144005.32	67858.111	106507.752	106133.743	0.065	139548.05	80858.086	108635.7	104248.871	0.298	0.391	0.96
unclassified_Lachnospiraceae	121909.8	57227.435	101746.216	70024.509	0.448	133989.325	65667.697	100663.35	68026.299	0.328	0.707	0.96
Klebsiella	193256.06	419923.89	55245.528	75469.547	0.229	247995.625	464244.012	52132.154	75324.513	0.837	0.55	0.966
Parabacteroides	46675.52	14835.245	111546.964	83863.954	0.065	78613.875	67891.536	104138.315	82734.404	0.576	0.391	0.96
Ruminococcus	103382.96	92351.635	82937.836	100321.389	0.188	150388.6	91483.574	76492.55	96615.572	0.044	0.55	0.96
Gemmiger	58292.96	66652.426	81340	66135.67	0.355	84090.3	109887.792	76484.754	59508.356	0.976	0.592	1
Agathobacter	60712.08	47552.218	77457.708	86279.579	0.787	57262.625	39869.534	77344.331	85488.355	0.837	0.914	0.966
Prevotella	46072.48	39573.764	42515.72	66717.837	0.3	24680.7	21797.787	45943.562	66379.145	0.883	0.563	0.981
unclassified_Caudoviricetes	60985.64	26366.943	65084.176	32835.66	0.872	63157.575	47996.628	64592.396	29535.756	0.702	0.914	0.966
Eubacterium	50618.14	44637.669	54245.152	47482.886	0.914	33704.8	27442.621	56707.704	48183.924	0.461	0.914	0.96
Blautia	55649.48	24190.472	34653.516	20566.158	0.057	49710.925	25129.769	36374.677	21741.787	0.198	0.391	0.96
unclassified_Viruses	54507.1	78740.885	30148.896	20393.954	0.914	18429.175	12594.554	36636.2	37751.583	0.245	0.914	0.96
Dialister	44782.56	90957.282	32642.112	62328.562	0.746	81087.675	88439.756	27523.65	61166.143	0.198	0.914	0.96

Note: Data are presented as mean ± standard deviation (SD) of absolute abundances (MetaPhlAn4-derived abundance scores, not percentages). Statistical comparisons were performed using Mann-Whitney *U* tests. *P_value.x* and *P_value.y* represent raw *P* values for comparisons between RF-positive vs. RF-negative, and anti-CCP-positive vs. anti-CCP-negative groups, respectively. RF_FDR and ACPA_FDR are Benjamini-Hochberg false discovery rate (FDR) *q*-values. Bold values indicate nominal significance ($P < 0.05$).
