# Supplementary material for: Metagenomic characterization of gut microbiota in rheumatoid arthritis-associated interstitial lung disease: taxonomic shifts and clinical correlations
Source: Front Immunol. 2026 Jun 12;17:1868704. doi: 10.3389/fimmu.2026.1868704 (PMC13303103; doi:10.3389/fimmu.2026.1868704)
Supplement: Supplementary file 10 [file Table6.pdf]

Supplementary Table S6. Complete list of feature importance scores from random forest classification (HC vs. RA)

Feature	Importance
Bifidobacterium	0.0910016613629584
unclassified_Oscillospiraceae	0.06737402088059169
unclassified_Lachnospiraceae	0.049222925496220954
Escherichia	0.04792978872037129
Klebsiella	0.04731534901948292
Prevotella	0.04375594671066806
unclassified_Enterobacteriaceae	0.035315645807550505
Eubacterium	0.03501940332394878
Ruminococcus	0.034588965113045995
Alistipes	0.03437902611716457
Phocaeicola	0.03437680514410618
Streptococcus	0.03174303409907477
unclassified	0.03097785976278623
unclassified_Bacteria	0.030714680763576436
unclassified_Bacteroidales	0.026970378046622386
unclassified_Bacteroidaceae	0.026639064198823555
Gemmiger	0.02633572559268463
unclassified_Eubacteriales	0.02571593903172851
Bacteroides	0.025443983249598873
simpson	0.024459041421541424
unclassified_Caudoviricetes	0.023442001319758675
Clostridium	0.022533634054945784
Roseburia	0.022133979380706915
unclassified_Clostridia	0.020121428922177487
Blautia	0.0194881477364398
Agathobacter	0.01931730469965764
unclassified_Prevotellaceae	0.017334753787878784
Segatella	0.017020451192326192
Parabacteroides	0.01500356125356125
Faecalibacterium	0.014183877020415481

Note: Feature importance was calculated using the random forest algorithm (1000 trees) based on genus-level relative abundances and alpha diversity indices. Importance is measured as the mean decrease in accuracy when the feature is permuted. Higher values indicate greater contribution to the classification of healthy controls (HC) versus rheumatoid arthritis (RA) patients. Model performance was assessed by 10-fold cross-validation (AUC = 0.96).
