# Supplementary material for: Metagenomic characterization of gut microbiota in rheumatoid arthritis-associated interstitial lung disease: taxonomic shifts and clinical correlations
Source: Front Immunol. 2026 Jun 12;17:1868704. doi: 10.3389/fimmu.2026.1868704 (PMC13303103; doi:10.3389/fimmu.2026.1868704)
Supplement: Supplementary file 11 [file Table7.pdf]

Supplementary Table S7. Complete list of feature importance scores from random forest classification (RA-ILD vs. RA)

Feature	Importance
unclassified_Bacteroidaceae	0.08869934652900463
Parabacteroides	0.0542878334214706
Blautia	0.0527973940289415
Phocaeicola	0.05100198560684243
Bacteroides	0.04768330456794218
Ruminococcus	0.041014791672251034
Bifidobacterium	0.04083655717204479
unclassified_Bacteria	0.03825440930410512
Escherichia	0.03471056651827398
unclassified	0.034206558413529195
Alistipes	0.03222965093444785
unclassified_Bacteroidales	0.03197417124069601
simpson	0.030791332284397125
unclassified_Lachnospiraceae	0.030681038829161896
Gemmiger	0.02920662536637769
Klebsiella	0.02640307343772454
Eubacterium	0.02634796019504804
unclassified_Enterobacteriaceae	0.0257142791087727
unclassified_Oscillospiraceae	0.025612080627705633
unclassified_Eubacteriales	0.024776108928419856
Streptococcus	0.024657315430670693
Segatella	0.023032675587103934
Clostridium	0.02247384828125282
unclassified_Prevotellaceae	0.02155783930531277
shannon	0.01829264059968717
Agathobacter	0.017905686869889654
unclassified_Caudoviricetes	0.01780392156862745
Prevotella	0.01678341786475356
unclassified_Bacillota	0.015084966081676605
unclassified_Clostridia	0.014808638663319046
Faecalibacterium	0.01452012019382651 8
Roseburia	0.013018990154601786

Note: Feature importance was calculated using the random forest algorithm (1000 trees) based on genus-level relative abundances and alpha diversity indices. Importance is measured as the mean decrease in accuracy when the feature is permuted. Higher values indicate greater contribution to the classification of rheumatoid arthritis-associated interstitial lung disease (RA-ILD) versus rheumatoid arthritis (RA) patients. Model performance was assessed by 10-fold cross-validation (accuracy = 82%).
