# Supplementary material for: Metagenomic characterization of gut microbiota in rheumatoid arthritis-associated interstitial lung disease: taxonomic shifts and clinical correlations
Source: Front Immunol. 2026 Jun 12;17:1868704. doi: 10.3389/fimmu.2026.1868704 (PMC13303103; doi:10.3389/fimmu.2026.1868704)
Supplement: Supplementary file 12 [file Table8.pdf]

Supplementary Table S8. Mean relative abundances of functional genera in HC, RA, and RA-ILD groups

Group	Bacteroides	Faecalibacterium	Escherichia	Prevotella	Roseburia	Lactobacillus	Bifidobacterium	Akkermansia	Clostridium	Ruminococcus
**HC**	9.344	8.657	3.072	2.447	1.063	0.029	1.192	0.391	1.505	1.517
**RA**	12.274	5.420	4.651	0.540	2.959	0.043	0.364	0.408	1.632	1.387
**RA-ILD**	13.591	8.463	5.153	1.003	1.722	0.049	0.245	0.373	1.907	1.182

Note: Data are presented as mean relative abundance (%) ± standard deviation (SD) for each group. The ten genera were selected based on established functional roles in inflammation and gut health. Group comparisons were performed using Kruskal- Wallis tests followed by Dunn's post- hoc tests with Benjamini- Hochberg correction (data not shown). The underlying sample- level data were used to generate Figure 6.
