# Supplementary material for: Metagenomic characterization of gut microbiota in rheumatoid arthritis-associated interstitial lung disease: taxonomic shifts and clinical correlations
Source: Front Immunol. 2026 Jun 12;17:1868704. doi: 10.3389/fimmu.2026.1868704 (PMC13303103; doi:10.3389/fimmu.2026.1868704)
Supplement: Supplementary file 13 [file Table9.pdf]

Supplementary Table S9. ROC analysis of microbial markers versus traditional inflammatory markers for distinguishing RA-ILD from RA-non-ILD.

Marker	AUC	95% CI	Sensitivity (%)	Specificity (%)	Cutoff
Escherichia/Shigella	0.825	0.662–0.988	80.0	80.0	245,000
Roseburia	0.810	0.652–0.968	70.0	85.0	50,000
CRP	0.540	0.340–0.740	50.0	60.0	30.0 mg/L
ESR	0.610	0.420–0.800	70.0	60.0	65.0 mm/h
Combined (Escherichia + Roseburia)	0.850	0.688–0.999	80.0	80.0	—
